# Age-Dependent Serum Volatilomics of Milk and Yogurt Intake: A Randomized Crossover Study in Healthy Young and Older Men

**DOI:** 10.1021/acs.jproteome.2c00674

**Published:** 2023-03-24

**Authors:** Hélène Yi Meng, Jinyoung Kim, Charlotte Fleuti, Pascal Fuchsmann, Sergio Polakof, Dominique Dardevet, Corinne Marmonier, Kathryn J. Burton-Pimentel, Ulrich Bütikofer, Guy Vergères

**Affiliations:** † Agroscope, Schwarzenburgstrasse 161, 3003 Bern, Switzerland; ‡ Unité de Nutrition Humaine (UNH), INRAE, Université Clermont Auvergne, F-63000 Clermont-Ferrand, France; § CNIEL, 42 Rue de Châteaudun, F-75009 Paris, France

**Keywords:** biomarker, volatilomics, VTT extraction, nutrition, plasma serum metabolome, yogurt, milk, age

## Abstract

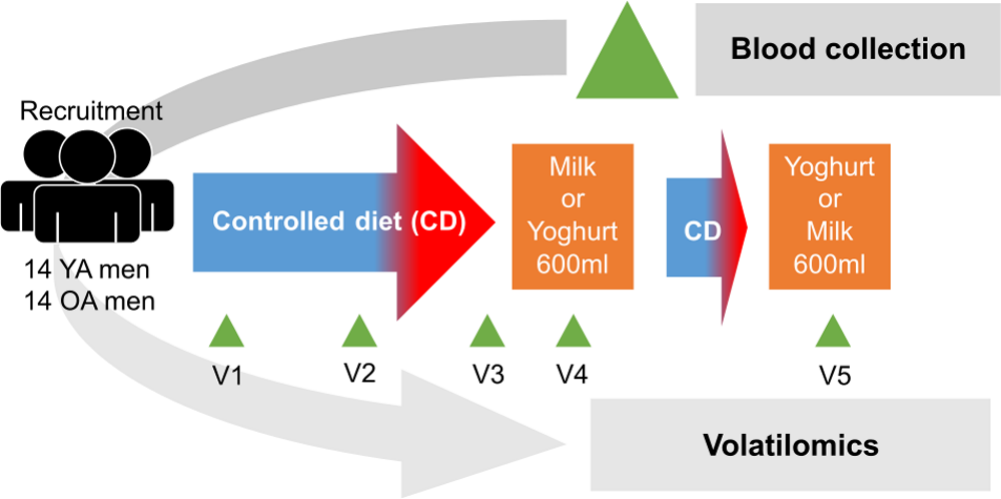

Nutritional biomarkers of dairy intake can be affected by both food transformation and the metabolic status of the consumer. To assess these effects, this study investigated the serum volatilome of 14 young (YA) and 14 older (OA) adult men undergoing a 3 week restriction of dairy and fermented foods followed by a randomized crossover acute intake of milk and yogurt. 3,5-Dimethyl-octan-2-one was identified as a potential marker of dairy product intake as its response after both milk and yogurt intake was significantly increased during the postprandial phase but significantly decreased in fasting serum samples of the OA group after the restriction phase. The postprandial response of two metabolites was significantly different for the two dairy products while 19 metabolites were modulated by age. Remarkably, the response of all age-dependent metabolites was higher in the OA than in the YA group after milk or yogurt intake, whereas at the end of the restriction phase, their fasting concentrations were lower in the OA than in the YA group. Among these, *p*-cresol, a specific marker of colonic protein fermentation, had a significant response in the OA but not the YA group, which may suggest impaired intestinal processing of dietary proteins in the OA group.

## Introduction

The complex and subtle nature of the interaction of foods with the human organism is a challenge in nutrition research that can be addressed, in part, through the use of ‘omics’ technologies that report on the dynamic response of the individual organism to the nutritional environment and ultimately promising the realization of personalized nutrition.^1,2^ Among the omics that report on molecular changes in the cell, metabolomics has emerged as a key tool in nutritional research because of the very obvious metabolic nature of nutrition, which has led to the notion of ‘nutrimetabolomics’.^3−6^

Dairy products constitute a substantial fraction of human diets. In the last decade, there has been increasing interest in the identification of food intake biomarkers (FIBs) using metabolomics approaches, in particular via analysis in human blood and urine of the postprandial response to dairy intake.^7−11^ Although studies are often conducted in healthy young men, the effect of age on food intake biomarkers has been identified as the physiological function and the metabolism have been shown to differ with aging,^12−15^ such as alterations in the gut microbiota implicating differences in the metabolic degradation of macro- and micronutrients.^16−20^ Several reports have included targeted analyses of the postprandial response of older adults to food intake^21−24^ or the identification of aging biomarkers in the fasting state.^25−27^ However, no study has investigated the impact of age on the analysis of biomarkers associated with the intake of dairy products.

Recently, Kim *et al.* conducted an intervention with young and older adult men designed to investigate the effects of a 3 week dietary restriction phase, excluding dairy products and reducing fermented foods, combined with two acute challenges, in a crossover design, with milk and yogurt.^28,29^ An untargeted metabolomics approach, with liquid chromatography (LC–MS) and gas chromatography (GC–MS), was taken to identify candidate markers associated with dairy product intake in the two study groups.^29^ This atypical study design combined a short-term dietary exclusion phase with acute challenges in two distinct age groups. Furthermore, the metabolomes of both human serum samples and dairy products were measured. This unique study design allowed for testing of the robustness of candidate dietary biomarkers.^30^

The aim of the present report is to make use of the above strengths of the study and to complement the report of Kim *et al.*(29) with an analysis of the volatile compounds (*i.e.*, the volatilome) of serum samples using dynamic headspace vacuum transfer in-trap (DHS-VTT) GC–MS.^31^ The volatilomics approach provides two specific advantages compared to the classical LC–MS and GC–MS methods. First, it extends the category of metabolites that can be measured and identified to volatile molecules of smaller size.^32^ Second, as the analysis of volatile compounds has a long and proven history in food science,^33−35^ volatilomics studies in nutrition offer the potential to strengthen the link between food composition, using food databases, and the human food metabolome. In this study, different types of biomarkers were investigated in the volatilome of serum samples, including those specific for (i) the intake of milk or yogurt (product effect); (ii) the intake of milk and yogurt (dairy effect); (iii) the effect of age on the response to dairy intake (dairy age effect) and to the short-term restriction of dairy products and fermented foods.

## Experimental Section

### Study Design

The samples and data for this investigation were collected for a dietary intervention study published in 2021 by Kim *et al.*(28) In total, 28 healthy men participated in this study (14 young adult (YA) men (median age of 27.5 y (Q1: 25.0, Q3: 31.0)) and 14 older adult (OA) men (median age of 69.0 y (Q1: 66.0, Q3: 71.0)). A summary of the cohort with the basic characteristics of the study population is given in Table 1.

**Table 1:** Clinical Parameters (Median Interquartile Range (IQR)) of Young Adult Men (YA) and Older Adult Men (OA) at Fasting at the Beginning of the Semi-controlled Diet (Adapted from Kim *et al.* (2021))^28^

	YA	OA
number of participants	14	14
age (years)	27.5 (25.0, 31.0)	69.0 (66.0, 71.0)
body weight (kg)	79.1 (75.1, 83.6)	71.4 (67.0, 74.9)
body mass index (BMI) (kg/m^2^)	25.1 (22.3, 25.9)	23.8 (22.6, 26.9)
insulin (pM)	26.32 (15.06, 33.93)	21.60 (14.34, 27.43)
glucose (mM)	5.31 (4.95, 5.56)	5.19 (5.03, 5.35)
triglycerides (mM)	0.67 (0.61, 1.06)	0.93 (0.74, 1.20)
total cholesterol (mM)	3.98 (3.59, 4.53)	5.06 (4.88, 5.84)
HDL (mM)	1.14 (1.01, 1.37)	1.21 (1.15, 1.41)
LDL (mM)	2.28 (2.06, 2.62)	3.36 (2.79, 3.56)

**Figure 1:**
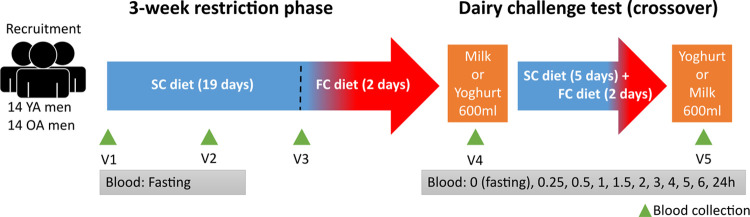
Schematic overview of the study design. Abbreviations: OA: older adult; YA: young adult; SC: semi-controlled; FC: fully controlled.

The participants were required to undergo a 3 week period (19 days) with a semi-controlled (SC) diet followed by 2 days with a fully controlled (FC) diet before the acute tests were conducted with dairy products (Figure 1).

For the semi-controlled diet, dairy products were eliminated and fermented foods were significantly reduced from the participants’ diets. The fully controlled diet was completely free of both dairy and fermented foods. The aim of the 3 week controlled phase was to reduce circulating concentrations of molecules associated with dairy and fermented food intake and to maximize the magnitude of participants’ postprandial response to the acute intake of dairy products. In addition, this phase supported the identification of metabolites that might respond to both dietary phases of the study design. Verification of the participants’ adherence to the controlled phase was performed as described by Kim *et al.*(28) Fasting serum samples were collected before (V1) and during the 3 week controlled phase (V2, V3). The dairy products were tested according to a crossover design requiring two acute intakes of milk and yogurt, with the order randomly assigned within each group so that all participants consumed the same study foods. Before the second day of acute intervention, the participants underwent a further week of the semi-controlled diet (as described above). As for the other intervention day, for the 2 days before the test, the diet of the participants was fully controlled.

On the day of the postprandial test response to the acute intake of dairy products, the participants consumed 600 mL of whole UHT-milk or yogurt as described in Kim *et al*.^29^ Serum samples were collected at several time points during the day (0 (overnight fasting, named V4 or V5 according to the first or second intervention day, respectively), 0.25, 0.5, 1, 1.5, 2, 3, 4, 5, 6, and 24 h).

### Chemicals

All required chemical compounds were purchased from Sigma-Aldrich Chemie GmbH (Buchs, Switzerland).

### Sample Logistics

**Figure 2:**
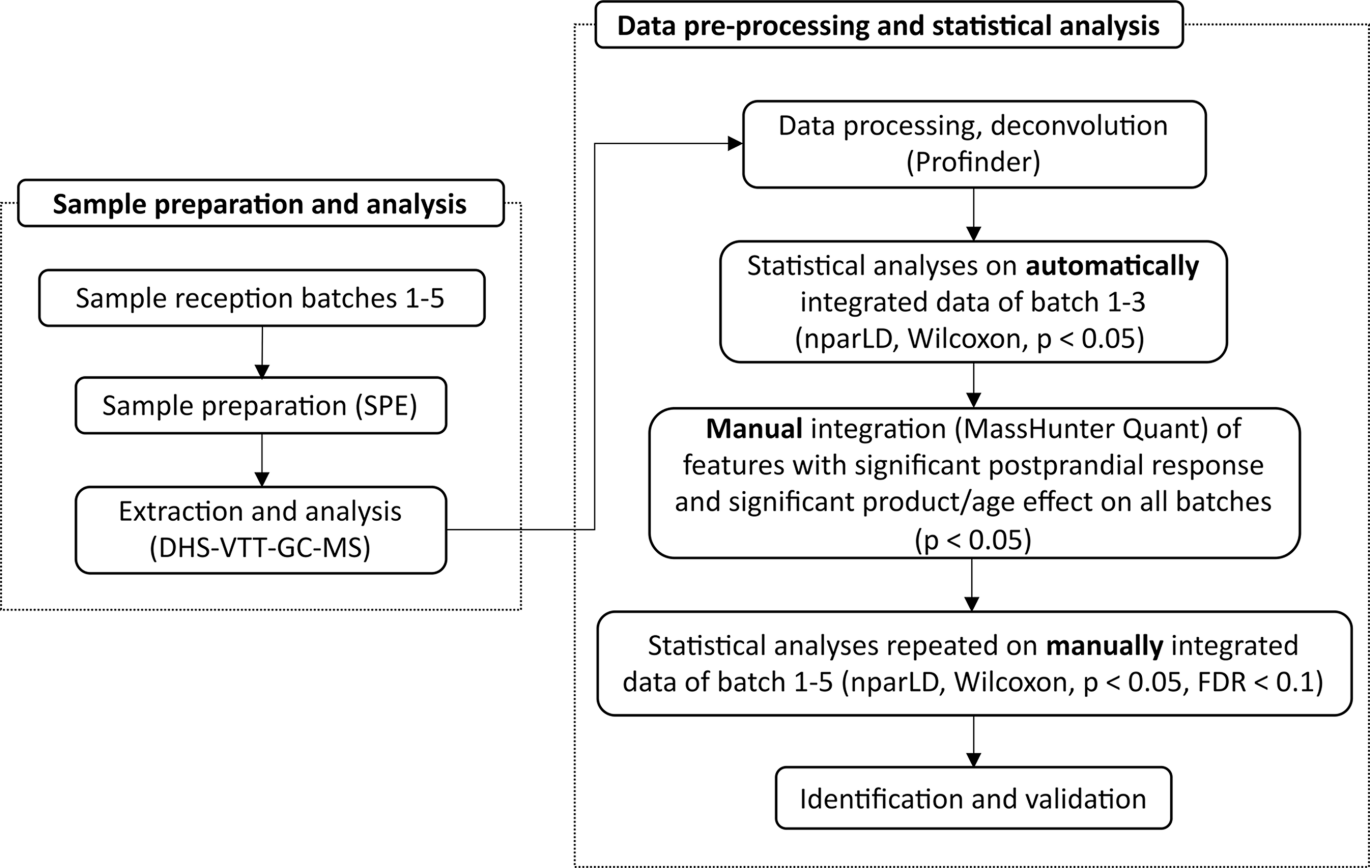
Overview of the sample preparation and data analysis strategy for serum samples. Abbreviations: DHS-VTT-GC-MS, dynamic headspace vacuum transfer in-trap gas chromatography coupled with mass spectrometry; FDR, false discovery rate; nparLD, nonparametric analysis of longitudinal data; SPE, solid phase extraction.

All samples were received over a period of 1 year in five batches. Each batch was measured shortly after reception of the samples. Before the start of the measurement, the samples were stored at −80 °C. The number of samples per batch were respectively 152, 100, 125, 200, and 125 for batches 1–5. A workflow summarizing the sample and data analysis is presented in Figure 2.

### Sample Preparation

The volatile compounds in serum were first concentrated using solid phase extraction (SPE) to remove the matrix effect. This concentration step also removes water from the samples; water hinders the extraction of the headspace and can lead to unintentional water (vapor) injection. SPE polymeric cartridges (CHROMABOND HR-X (hydrophobic polystyrene-divinylbenzene resin), 45 μm particle size, 1 mL/30 mg, Macherey-Nagel AG, Oensingen, Switzerland) were conditioned with hexane 3 × 1 mL, acetonitrile 3 × 1 mL, nanopure water 3 × 1 mL, and H_3_PO_4_ 4% 1 × 1 mL; 450 μL of serum were mixed with 900 μL of internal standard solution containing 100 μg/kg hexanal-*d*_12_ and 100 μg/kg octanoic acid-*d*_15_ in H_3_PO_4_ 4%. The samples were passed through the conditioned SPE and dried under nitrogen for 20 min. The volatiles were then extracted by adding 600 μL of acetonitrile to the cartridge, and the extracts were collected in 2 mL glass vials. Samples were stored at −80 °C until analysis. Before GC–MS analysis, 100 μL of the extracted sample was pipetted in 20 mL headspace vials hermetically sealed with a septum silicone Teflon (Macherey-Nagel AG, Switzerland).

In order to associate candidate biomarkers of dairy intake to molecules present in the food products, yogurt and milk samples were prepared and analyzed in the same manner as serum samples: 5 mL of milk or yogurt was mixed with 5 mL of nanopure water and vortexed in Falcon tubes. The samples were then centrifuged (10 °C, 10 min, 2400 rpm); 5 mL of supernatants (without the cream for the milk samples) were collected and frozen until preparation by SPE. The processing of samples on SPE was the same as that for serum.

Quality control (QC) samples were prepared by combining 250 μL of serum of each sample from batch 1 (162 serum samples). The volatile compounds from the QC samples were extracted by SPE following the sample preparation procedure for serum.

### Sample Analysis

#### Extraction Parameters

The volatile compounds were extracted by dynamic headspace vacuum transfer in trap extraction (DHS-VTT)^31^ and analyzed by gas-chromatography mass spectrometry (GC–MS). The volatile compounds were adsorbed on a Tenax TA, 80/100 mesh (2/3 bottom)/Carbosieve S III, 60/80 mesh (1/3 top) ITEX (in-tube extraction) trap (BGB Analytik AG, Böckten, Switzerland) conditioned according to the supplier’s temperature recommendations (320 °C for 1 h) under a nitrogen stream of 100 mL min^–1^. Incubation of the samples lasted 5 min at 60 °C. Volatile compounds were extracted for 5 min at 5 mbar using a vacuum pump Buchi V-300 (Büchi, Flawil, Switzerland) with the syringe temperature during extraction set at 100 °C and the ITEX trap at 35 °C. Bound volatile compounds were desorbed for 2 min with a nitrogen flow of 100 mL min^–1^ at 240 °C in a programmed temperature vaporizer (PTV) injector of type CIS4 (Gerstel AG, Sursee, Switzerland) in vent mode at 50 mL min^–1^ and 0 kPa for 30 s. The injector contained Tenax TA liner, which was cooled to 10 °C using liquid nitrogen to trap the compounds again. The injector was then heated at a rate of 12 °C s^–1^ to 240 °C to release the bound volatile compounds. After injection, the trap was reconditioned according to the supplier’s temperature recommendation (300 °C) for 15 min under a nitrogen flow of 100 mL min^–1^.

#### GC–MS Parameters

The analyses were completed using an MPS2 autosampler (Gerstel AG) on an Agilent 7890B GC system coupled to an Agilent 5977B mass selective detector (MSD) equipped with the High Efficiency Source (HES) (Agilent Technologies, Basel, Switzerland). Volatile compounds were separated on an OPTIMA FFAPplus fused silica capillary column (polyethylene glycol nitroterephthalate, cross-linked, 60 m × 0.25 mm × 0.5 μm film; MACHEREY-NAGEL, Oensingen, Switzerland) with helium as the carrier gas at a constant flow of 1 mL min^–1^ (25.641 cm s^–1^). The oven temperature was programmed as follows: 5 min at 40 °C and then heated to 220 °C at a rate of 5 °C min^–1^ with a final hold time of 20 min, to make a total run time of 61 min.

The MS settings were as follows: the transfer line was fixed at 250 °C and the source temperature at 230 °C; the analytes were monitored in SCAN mode between 30 and 350 amu with a 7 min solvent delay; the MS detector was switched off between 15.7 and 17.2 min to cut off the solvent peak; the gain was set at 15. The autosampler was controlled with a Cycle Composer V.1.5.4 (CTC Analytics, Zwingen, Switzerland) and PTV injector with Maestro1 software V.1.4.8.14/3.5 (Gerstel AG).

### Data Pre-processing and Statistical Analysis

#### Reduction and Filtering of Serum Datasets

MS signal deconvolution and grouping were achieved using Masshunter Profinder software version 10.0 (Agilent Technology, Santa Clara, CA, USA). Agile 2 was used for performing deconvolution. Metabolites with a signal less than three times the median height of the background noise were excluded during the deconvolution. Due to a change of the electron multiplier and a leak in the MS system between batches 3 and 4, a significant shift of the peaks was observed between batches 1–3 and 4–5. The automatic deconvolution was therefore performed only on batches 1–3, as Profinder does not allow peak alignment for GC–MS data. The full data matrix of automatically deconvoluted features of batches 1–3 is available as in the Supporting Information (Table S2).

### Statistical Analysis

Statistical analyses were processed by R (v.4.0.3; R Foundation for Statistical Computing, Vienna, Austria) following the workflow presented by Kim *et al*.^29^ for untargeted LC–MS analyses. Using the dataset obtained after automatic deconvolution of batches 1–3, grouping, and standardization of the signals using the hexanal-*d*_12_ area, nonparametric analysis of longitudinal data with the ld.f1 function was used to determine whether a feature had a significant postprandial response during the acute intake of milk and yogurt (nparLD, *p* < 0.05). The incremental area under the curve (iAUC) (MESS package v0.3.2) of the features selected by the nparLD test was further tested by the Wilcoxon signed-rank test (*p* < 0.05) to determine if each was significantly different from zero in at least one of the four groups (young adults after milk intake: YA-M; young adults after yogurt intake: YA-Y; older adults after milk intake: OA-M; older adults after yogurt intake: OA-Y). The iAUC is a simple and robust metric that allows characterization of relative changes in the concentration of metabolites of interest in the acute response to food intake.^36,37^

To investigate age and product effects, the previously selected features with significant postprandial response were tested by f1.ld.f1 using the iAUCs (nparLD, p < 0.05). If a feature showed significant differences in the acute response between the YA and OA groups (age effect) or after milk or yogurt intake (product effect), a Wilcoxon signed-rank test was applied to identify which of the groups responded significantly: a feature with a significant product effect was confirmed by the test in the separate age group (YA-M *vs* YA-Y and OA-M *vs* OA-Y, paired, p < 0.05), and those with a significant age effect was confirmed by the test in the separate product group (YA-M *vs* OA-M and YA-Y *vs* OA-Y, non-paired, *p* < 0.05).

The features showing a significant product and/or age effect were then manually integrated with MassHunter Quantitative Analysis (Agilent Technology, v10.1) on all batches to remove analytical false positives, and signal drift was corrected via the QC-based robust locally estimated scatterplot smoothing signal (LOESS) correction method^38^ using R. 3,5-Dimethyloctan-2-one, a previously reported candidate marker of the acute intake of dairy products identified by untargeted volatilomics,^32^ was added to the list of molecules to be characterized after manual integration. The manually integrated features were re-analyzed statistically following the same treatments as above, and those that remained significant were maintained for further analysis and identification. The full data matrix of the manually integrated features is available in the Supporting Information as Table S3.

To test the behavior of the above molecules during the 3 week restriction phase eliminating dairy products and significantly reducing fermented foods, the manually integrated postprandial features were tested for treatment (*i.e.*, 3 week restriction) and age effects with the f1.ld and f1.ld.f1 functions respectively (nparLD, p < 0.05). The age effect was tested for V1, V2, V3, and V4 comparing both age groups with the Wilcoxon signed-rank test. For the effect of the 3 week restriction, V1 was tested against V4 (Wilcoxon signed-rank test, *p* < 0.05). If the iAUC of a feature was significantly positive after acute milk and yogurt intake in the YA or OA group and its intensity at V4 sampling at the end of the 3 week restriction period was significantly lower than at V1 sampling at the beginning of the 3 week restriction period, the feature was considered to have potential as a marker of dairy intake beyond acute (postprandial) conditions. Benjamini–Hochberg false discovery rate (FDR) correction was applied to the manually integrated dataset (*p* < 0.05 and FDR < 0.1).

Postprandial kinetics were visualized using heatmaps generated with hcluster from the R package amap (version 0.8-19).

### Identification of Metabolites

The identification of metabolites was carried out taking into account the correspondence (Match factor) with the NIST database (The National Institute of Standards and Technology NIST/EPA/NIH mass spectral library (NIST17) version 2.3 (NIST, Gaithersburg, MD, USA)) as well as the retention index (RI), determined using an injected alkane series (C7:C40) and the injection of the pure standard if possible. The pure standard was dissolved in either methanol or hexane and then extracted by DHS-VTT. The parameters used for the analysis of the standards and the alkane series were those used for GC–MS. Based on the identification criteria recommended by the Metabolomics Standards Initiative (MSI),^39,40^ the metabolites are classified according to 4 confidence levels (1–4), level 1 being the most exigent level.

Level 1: identification using the injection of the pure standard and the comparison of the spectrum obtained with a database (minimal match factor of 90%) and the RI (maximal relative difference of about ±10–15).

Level 2: identification to the spectrum of the database with a match factor of >80% and a maximal relative difference in RI of ±15.

Level 3: implies that the compound has a putative attribute of a compound class and has physicochemical properties and spectral similitude consistent with a compound from a reference library.

Level 4: corresponds to unknown compounds. The compound cannot be identified with the databases or the RI.

## Results and Discussion

### Overall Characteristics of the Volatile Postprandial Serum Metabolome

**Figure 3:**
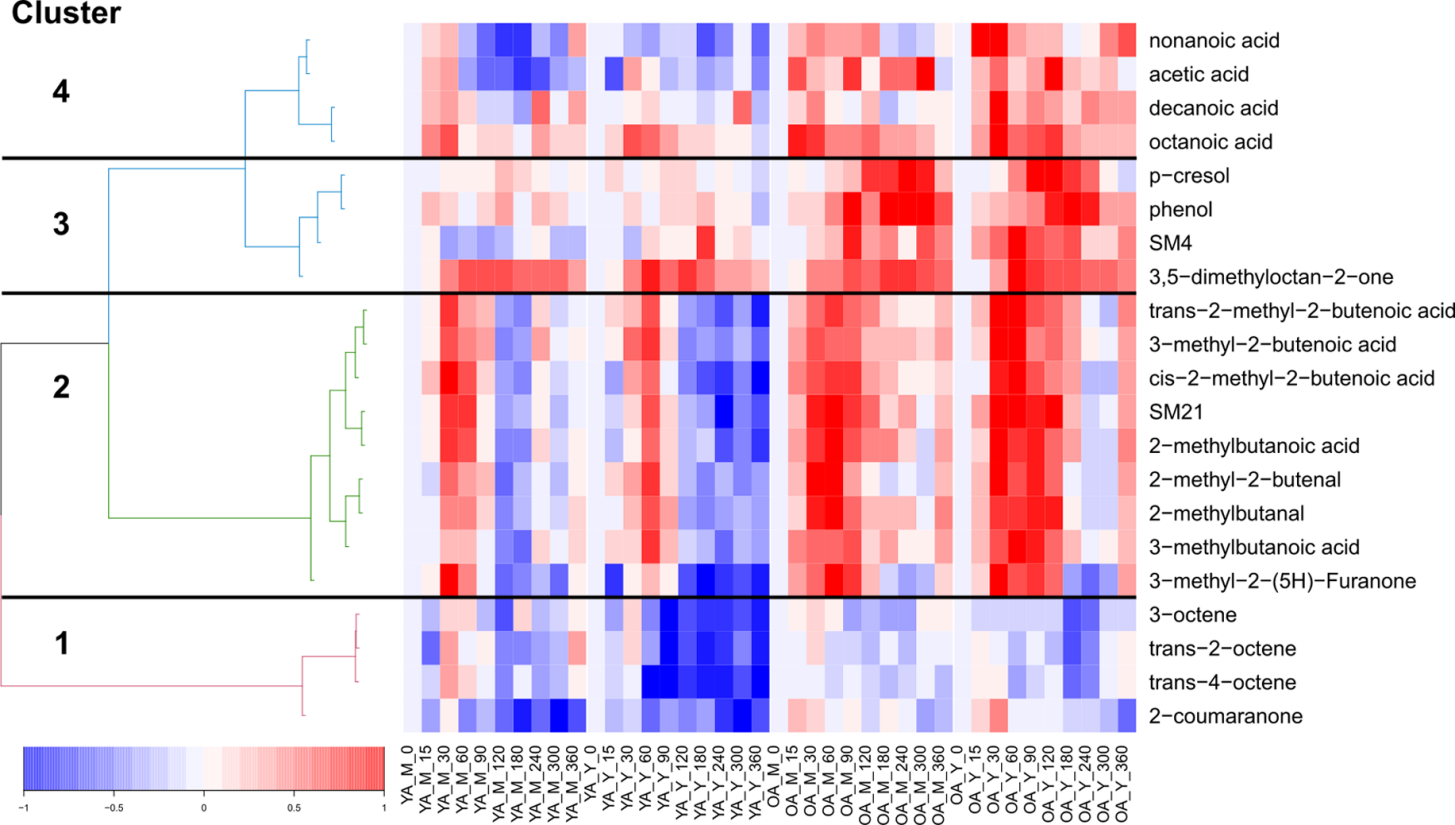
Heatmap of 21 metabolites with significant postprandial response and significant age or product effects. The fasting (zero) time point is subtracted for each time point. Normalization to 1 was performed with the highest absolute value of the metabolite. Clustering of metabolites was conducted with hcluster (method = Euclidean, link = ward) The labeling of each sample on the horizontal axis provides the age group (YA: young adult, OA: older adult), product type (M: milk, Y: yogurt), and postprandial time point (in minutes).

In serum samples, a total of 8227 features were detected after data pre-processing by untargeted DHS-VTT-GC–MS. In all, 808 features showed a significant postprandial response (ld.f1: *p* < 0.05; iAUC ≠ 0: *p* < 0.05), corresponding to 10% of the total detected features. A total of 21 metabolites with significant postprandial response and significant age or product effects are reported in Table 2A. A heatmap with all 21 metabolites showing their kinetics is given in Figure 3. The heatmap was divided into four main clusters, according to the distance of the clusters. Boxplots with the iAUCs and the significant effects are shown in Figure S1.

**Table 2A:** List of Identified Serum Metabolites with a Significant iAUC Combined with a Product or Age Effect^a^

							occurrence in dairy products		postprandial effect	
serum metabolite (SM) number	potential identification	functional group	fragment used (*m*/*z*)	RI sample	RI standard injection	identification level	milk	yogurt	product effect (*p* < 0.05)	age effect (*p* < 0.05, FDR < 0.01)
SM1	2-methylbutanal	aldehyde	86	935	935	1	x	x		x
SM2	2-methyl-2-butenal		84	1132	1129	1	x	x		x
SM3	3,5-dimethyloctan-2-one	ketone	72	1383	1375	1				
SM4	furanone derivative		126	1750	NA	3				x
SM5	3-methyl-2-(5*H*)-Furanone		98	1805	1800	1	x			x
SM6	2-coumaranone		134	2180	2173	1		x		x
SM7	phenol	benzenoid	55	2061	2056	1	x	x		x
SM8	*p*-cresol		81	2140	2137	1	x	x		x
SM9	acetic acid	fatty acid	62	1477	1482	1	x	x		x
SM10	2-methylbutanoic acid		87	1693	1689	1	x	x		x
SM11	3-methylbutanoic acid		103	1694	1696	1	x	x		x
SM12	*cis*-2-methyl-2-butenoic acid		82	1807	1812	1				x
SM13	3-methyl-2-butenoic acid		82	1833	1833	1		x		x
SM14	*trans*-2-methyl-2-butenoic acid		82	1882	1880	1		x		x
SM15	octanoic acid		83	2085	2088	1	x	x		x
SM16	nonanoic acid		129	2192	2193	1	x	x		x
SM17	decanoic acid		129	2299	2299	1	x	x		x
SM18	*trans*-4-octene	hydrocarbon	112	839	836	1				x
SM19	*trans*-2-octene		112	864	864	1	x	x	x	
SM20	3-octene		42	875	873	1	x	x	x	x
SM21	unknown	unknown	75	1695	NA	4				x

a Significant (*p* < 0.05) positive difference in blue and significant negative difference in green. NA = not applicable.

Nineteen features (corresponding to 2.4% of the postprandial features) were found to differ in their response in the two age groups (Table 2A, Figure S1). Two features, corresponding to 0.2% of the postprandial features, differed between the product groups (Table 2A, Figure S1). The postprandial kinetics of these molecules are shown in Figure S2. The mass spectra of non-identified features can also be found in the Supporting Information (Figure S3).

### Postprandial Product Effect in Serum

Two postprandial serum metabolites (*trans*-2-octene and 3-octene) showed a product effect (Table 2A, Figure S1) with a significantly higher iAUC in the YA-M group compared to the YA-Y group (f1.ld.f1 and Wilcoxon, *p* < 0.05). However, these differences were not significant after FDR correction. These two compounds were placed in cluster 1 in the heatmap (Figure 3), a cluster which demonstrates short postprandial responses before return to baseline. These molecules were also detected in both dairy products. Although these compounds were previously identified in adult stools,^41^ to the best of our knowledge, this is the first report of their presence in human serum.

In this study, few metabolites could differentiate milk from yogurt intake. As mentioned by Kim *et al*.,^29^ this could be due to the fact that, in contrast to *Streptococcus thermophilus*, *Lactococcus delbrueckii* subsp. *bulgaricus* was only present in very low numbers in the mild bacterial starter culture used to produce our yogurt and, additionally, was not able to grow in either milk or a culture media specific for this species. Changes in the composition of milk upon fermentation to yogurt were thus largely limited to the action of *S. thermophilus,* which might have decreased the number of postprandial metabolites that differentiated the intake of the two products.

### Postprandial Dairy Effect in the Serum Volatilome

**Figure 4:**
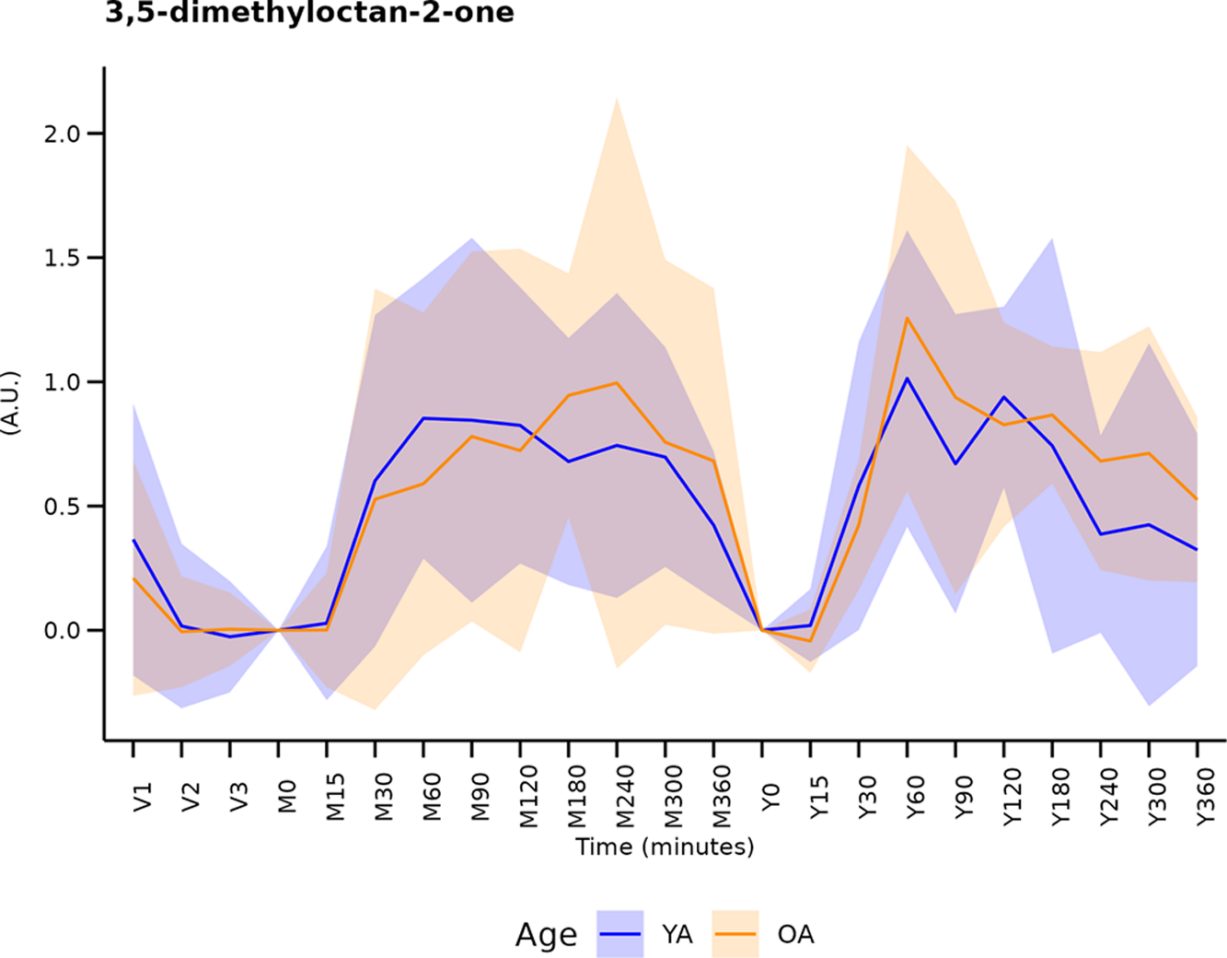
Relative concentrations of 3,5-dimethyloctan-3-one, a candidate biomarker of dairy products, during the 3 week restricted period (V1, V2, V3) and during the 6 h postprandial test after milk (M0, M15, M30, M60, M90, M120, M180, M240, M300, M360) and yogurt (Y0, Y15, Y30, Y60, Y90, Y120, Y180, Y240, Y300, Y360) challenge. YA: young adults, OA: older adults.

While not present in dairy products, 3,5-dimethyloctan-2-one was previously shown to be specifically increased in serum after intake of milk and cheese but not a soy-based drink.^32^ This made it an interesting molecule to target in this study in order to further evaluate its potential as a marker of dairy product intake. The inclusion of a restrictive dietary phase in this study particularly allows us to observe the behavior of this compound after removal of dairy products. A clear postprandial signal for 3,5-dimethyloctan-2-one was observed after milk and yogurt intake by both the YA and OA groups in our study (Figure 4).

Interestingly, no age or product effect was observed for the postprandial behavior of this molecule. However, the restriction phase resulted in a decrease in the fasting levels of 3,5-dimethyloctan-2-one, that was only significant in the OA group, although the *p* value of the Wilcoxon test for the YA group was 0.058 (Table S1). Taken together, these results indicate that 3,5-dimethyloctan-2-one could be a robust marker for the intake of dairy products in a broad range of conditions. Of note, the molecule could not be detected in the dairy products in agreement with Fuchsmann *et al.*(32) Identifying the parent molecule, evidently a fat-derived molecule, in dairy products leading to the production of 3,5-dimethyloctan-2-one by the human organism will be key in validating this molecule as a plausible biomarker of intake.^42^

In this study, we also evaluated the dairy effect among the 21 identified metabolites demonstrating a postprandial change combined with an age or product effect. Only octanoic acid had a significant postprandial response after milk and yogurt intake and showed a change during the 3 week restriction phase in the OA group. In other words, the postprandial increases in octanoic acid after milk and yogurt intake were preceded by a significant decrease in their fasting levels during the 3 week restriction phase (see Figure S2, octanoic acid). Apart from octanoic acid, identification of the pool of metabolites that increased postprandially after the intake of dairy products (*e.g.,* milk and/or yogurt), independently of an age or product effect, as well as decreased during the 3 week restriction phase would have required a change in the analytical workflow with an initial manual integration, in each of the 702 serum samples, of all 808 features demonstrating a postprandial response after the automatic integration. This task was beyond the available resources and consequently not conducted.

Interestingly, octanoic acid not only demonstrated a significant dairy effect in the OA group, but also an age effect was observed both after milk and yogurt intake (see Table 2B, Figure S2). This led us to investigate the postprandial age effect after milk or yogurt intake.

**Table 2B:**
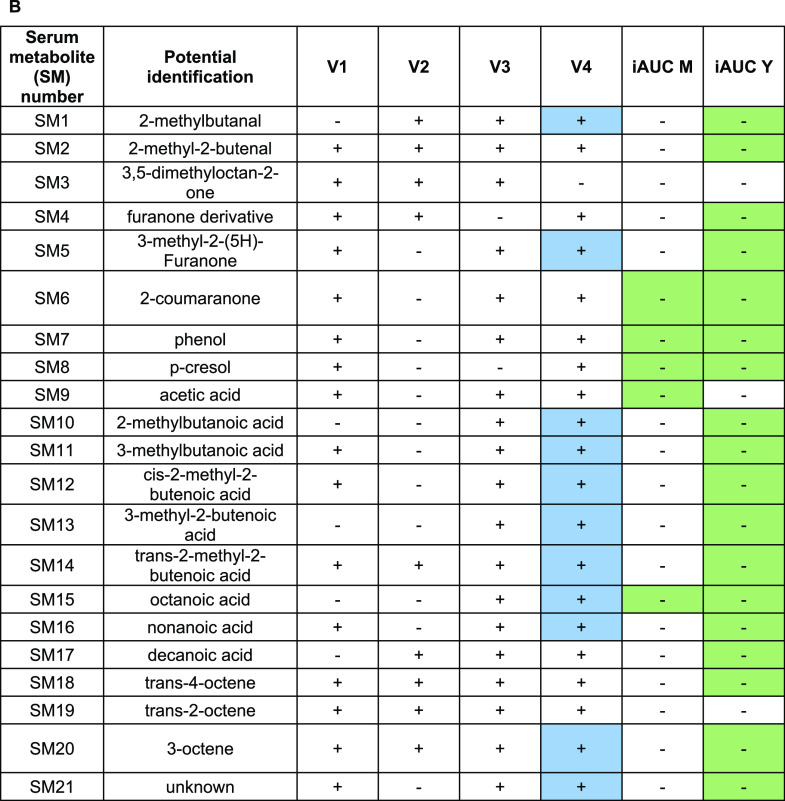
Positive (+) or Negative (−) Differences in Median Values of Each Visit (V1, V2, V3, V4) and of iAUC after Milk and Yogurt Intake (iAUC M, iAUC Y) between Young and Older Adult Groups for Each Serum Metabolite^a^

a Significant (*p* < 0.05) positive difference in blue and significant negative difference in green. NA = not applicable.

### Postprandial Age Effect in the Serum Volatilome

All of the 19 serum metabolites detected with a significant difference between the OA and YA groups were characterized by a higher postprandial response for older men than young men (see Table 2B; Figure 3; Figure S1). Among these metabolites, 14 were associated with a significant age effect only after yogurt intake, whereas one metabolite showed an age effect only after milk intake. For four metabolites, an increased iAUC was observed in the OA group, compared to the YA group, after the intake of both milk and yogurt.

An age effect was observed for the postprandial response of nine fatty acids. The response for acetic acid was different between the age groups only after milk intake. Conversely, age-specific responses were only observed for 3-methylbutanoic acid, 2-methyl-2-butenoic acids (*trans* and *cis*), 3-methyl-2-butenoic acid, 2-methylbutanoic acid, nonanoic acid, and decanoic acid after yogurt intake. Octanoic acid showed an age effect after milk and yogurt intake (Figure S1). All these fatty acids were also detected in the milk and yogurt samples, with the exception of *cis*-2-methyl-2-butenoic acid and *trans*-2-methyl-2-butenoic acid, which were detected only in yogurt. Interestingly, the cluster analysis separated the fatty acids into two distinct clusters: clusters 2 and 4 (Figure 3). Cluster 2 contains branched-chain fatty acids, which tend to a slower postprandial return to baseline in the OA group. Cluster 4 includes the four unbranched fatty acids (acetic acid, octanoic acid, nonanoic acid, and decanoic acid). As with the branched-chain fatty acids, similar kinetics were observed in the OA group for the unbranched fatty acids.

Numerous studies have shown that short-chain fatty acids, mainly produced from dietary fibers by the gut microbiota, have beneficiary effects, in particular through the improvement of gut barrier function and the reduction of intestinal inflammation.^43−45^ Branched-chain fatty acids and medium-chain fatty acids were also shown to exert health benefits.^44,46,47^ In the context of these functional properties, the different postprandial responses observed with fatty acids in the YA and OA groups raise the interesting possibility of different dietary management by age group to modulate the circulatory levels of these metabolites, in particular with yogurt as an age effect was mainly observed after intake of yogurt.

Cluster 3 is characterized by two phenolytic compounds, namely, phenol and *p*-cresol, that were significantly increased (iAUC) after intake of both dairy product in the OA group but not in the YA group. The fasting concentrations of these molecules was not changed significantly during the restriction phase. However, both molecules were detected in the dairy products and they have previously been described as imparting typical barnyard aromas to such products.^48^ It is important to note that the postprandial increase in *p*-cresol we observed is not specific to the intake of dairy products, as we recently shown that this molecule also increases significantly after the intake of a non-dairy high-fat meal.^49^ Furthermore, cresol and phenol are products of the colonic fermentation of dietary proteins that are not produced by the human organism and can be indicative of a protein overload of the small intestine digestive capacity.^50^

The significantly higher postprandial increase in *p*-cresol and phenol in the OA population after an identical dairy challenge as the YA is remarkable and may be suggestive of a reduced capacity to digest and assimilate dietary proteins in the small intestine and/or of a difference in the composition of the gut microbiota. Whereas changes to composition of the gut microbiota during the aging process have been reported,^51^ evidence for an impaired intestinal absorption capacity in elderly individuals is notably scarce.^52^ Lancha *et al.*(53) suggested that the intake of approximately 0.4 g protein/kg BW per meal (*e.g.,* 24 g for a person weighing 60 kg) should be recommended for the supplementation of dietary proteins in the elderly population to limit muscle mass loss, noting that, in the context of the transfer of protein to the colon with potential deleterious effects, “the maximal intake of protein with no adverse effect is not known”. In our study, the participants were challenged with 600 g of milk or yogurt that contained 20 g of proteins, corresponding to the amounts recommended for elderly adults by Lanch *et al.* to limit muscle mass loss^53^ as well as by French dietary guidelines (>1 g/kg d).

Given the large interindividual variability observed in the postprandial appearance of *p*-cresol and phenol in the urine of the OA group (see Figure S1) and the difference between the YA and OA groups, the evaluation of postprandial *p*-cresol and phenol might contribute to better dietary management for the elderly population, in particular regarding protein intake.

The flavonoid 2-coumaranone was associated with a postprandial age effect. Although the metabolite could be detected only in yogurt, it was present in the serum both after milk and yogurt intake. 2-Coumaranone has been previously identified in *Moringa oleifera* leaves^54^ and in plasma samples in subjects with acute hepatic ischemia, where 2-coumaranone concentrations decreased for patients with hepatic ischemia,^55^ but it has not yet been linked to dairy products to the best of our knowledge. 2-Coumaranone was clustered in Figure 3 as cluster 1 together with the hydrocarbons (*trans*-2-octene, *trans*-4-octene, and 3-octene).

Older adults release higher incremental levels of 18 metabolites, among these branched-chain fatty acids, than young adults in the bloodstream after yogurt intake whereas this was the case for only 5 metabolites after milk intake. This effect could potentially be explained by differences in the food matrix: intestinal metabolite uptake can indeed be influenced by changes in the microstructure or physicochemical properties (*e.g.,* pH) of foods, such as the one induced by fermentation.^56,57^

### Age Modulates Short-Term Effects of Dietary Restrictions in the Serum Volatilome

A statistical analysis of age-dependency during the 3 week restriction phase of the aforementioned 21 metabolites revealed an interesting pattern. Indeed, concentrations of most of these metabolites were reduced in the fasting samples at the end of the 3 week restriction phase (V4) in the OA group compared to the YA group (see Table 2B). This difference in fasting values between the age groups was not observed at the beginning of the restriction phase (V1 & V2) nor at the beginning of the 2 day fully controlled phase (V3). These results suggest that the 2-day fully controlled diet was effective in reducing basal levels of potential markers of dairy intake, although only in the OA group. The lower fasting levels in the OA group also suggest that this group was either more susceptible to the impact of the dietary restrictions on the fasting metabolome or that the dietary restrictions were followed differently between both age groups.^28^

Remarkably, as described in the previous section, the iAUCs of most of the metabolites reported in Table 2A were higher in the OA group than in the YA group. Thus, lower concentrations of these metabolites for the OA group were observed under fasting conditions but higher amounts were produced after acute intake of the dairy products, in particular yogurt. Although a significant difference in adherence to dietary protocols between the two age groups was not observed, dietary fat intake was significantly lower in the YA group than in the OA group, resulting in a lower total energy intake in the former group despite higher protein intake.^28^ The reaction of the volatile compounds during the restriction phase and the postprandial phase can therefore potentially be due to the different intake of macronutrients. However, this behavior may also be indicative of different metabolic flexibility between the two age groups, as was previously reported for lean and obese subjects in response to a high-fat meal^58^ and reviewed by Lépine *et al*.^59^ Nevertheless, the differential effect reported here for the YA and OA groups is opposite to what might be expected: indeed, in light of the decreased metabolic flexibility associated with age,^60^ smaller iAUCs in the OA group would be expected in response to the dairy challenge. Another explanation could be the ability of the YA group to better resist in the restriction phase and better adapt to the challenge, therefore maintaining steadier levels of metabolites. On the other hand, Emerson *et al.*(61) concluded that there is inherently diminished metabolic capacity with aging, although the YA group in their study also appeared to respond with a lower iAUC for triglycerides in response to a high-fat meal. Although they are numerous alterations of digestive functions associated with the aging process, these changes are subtle and insufficiently characterized.^34^ In this context, our results indicate that the postprandial volatilome could be a sensitive source of biomarkers to investigate the impact of age on the metabolism as well as the underlying mechanisms.

### Strengths and Limitations

Currently, there is limited knowledge on the effect of age on the behavior of nutritional markers, in particular biomarkers of food intake. Our study showed that the age factor should be considered while investigating these candidate markers, in particular for their validation using more quantitative measurements, for example, for dose–response studies. The use of the DHS-VTT for the analysis of volatile compounds is also a strength of this study as it allowed preservation of the integrity of the samples while allowing the extraction of a wide range of volatile compounds compared to other headspace extraction methods.^31^ Another strength of this study is the combination of a controlled dietary protocol during the restriction phase with an acute intake with dairy products, providing additional evidence for the identification of specific metabolites – such as octanoic acid and 3,5-dimethyloctan-2-one – as markers of the intake of dairy products.

This study also presents some limitations. First, the small number of participants limited the results of the statistical analyses. Though a total of 28 participants were recruited, each group consisted of only 14 participants (14 YA-M and YA-Y, 14 OA-M and OA-Y). In particular, an effect of the 3 week restriction phase was observed only in the OA group, though visually we could observe consistent trends between V1 and V4 in the YA group as well (*e.g.*, Figure S1). If the study were to be conducted with a larger group of participants, these metabolites could potentially be significantly different after such a dietary restriction. With 2 × 14 subjects, this study was primarily designed to detect a large number of postprandial metabolites; the untargeted identification of candidate markers of intake on fasting samples during longer interventions might indeed require a larger number of subjects. Second, serum metabolites were trapped on an SPE cartridge to remove any matrix effects and were acidified to maximize the sensitivity of the fatty acids present in the samples. Although neutral and acidic compounds can still be extracted under acidic conditions, certain groups of molecules, specifically basic compounds, could potentially not have been extracted during sample preparation. Third, due to potential batch effects during the analysis (change of electron multiplier of the MS, shift in the retention indices), high variability between the samples could occur, which could possibly reduce the number of metabolites detected automatically by the deconvolution and grouping software. Fourth, in addition to age, gender is an important factor that could modify the metabolic response. Only men here were selected for this study to reduce variability of the metabolism, and including women in the future would evidently improve the representativeness of candidate biomarkers. We finally note that, in complement to the univariate approach taken in this study, a multivariate analysis of the data was not conducted to extract additional features with a postprandial product- or age effect due to the relative inaccuracy of the automatic feature extraction and integration and the consequent need to confirm the statistical significance with univariate analysis of the individual features after manual integration of their peak intensity. Whereas multivariate analyses can exploit variable correlations and identify hidden structures in data derived from complex biological systems,^4^ the relative advantages and disadvantages of univariate and multivariate analyses remain debated.^62^

## Conclusions

The volatile fraction of human biological tissues and fluids was, until recently, little researched in human nutrition studies. Recent progress in volatile extraction and identification has allowed expansion of current knowledge of postprandial metabolomes on its volatile component. Many of the postprandial metabolites reported in serum in this study were also detected in the dairy products, suggesting that, due to their small size and fast absorption, these molecules are not, or only partly, transformed by the human organism. On the other hand, this study further strengthened the use of 3,5-dimethyl-octan-2-one as a candidate marker of dairy product intake although this molecule is evidently not present in dairy products but, rather, metabolized from an unknown dairy fat precursor by the human organism. Independent of the fate of food-borne metabolites, untargeted volatilome analysis is emerging as an interesting analytical strategy to detect biomarkers of food intake.

Surprisingly, analysis of the serum volatilome of the study samples provided limited evidence for metabolites whose postprandial response is indicative of the fermentative status of the ingested dairy product. Indeed, Fuchsmann *et al.*(32) identified three discriminant volatile compounds as candidate markers of dairy intake, among which two were specific to cheese intake. However, yogurt is a product less fermented than cheese, and the fermentation of the particular yogurt in this study was reduced due to the fact that the specific strains of *L. delbrueckii* subsp. *bulgaricus* used in this study did not grow in the dairy matrix.^29^

The most remarkable observation resulting from the analysis of the volatilome is the age dependency of the metabolites reacting to the nutrition interventions during the postprandial phase as well as during the 3 week restriction period. Independent of the involved mechanisms, our results demonstrate that lower fasting values of metabolites that are acutely reactive to food intake are generally associated with higher postprandial response in older adults.

## Supporting Information Available

Table S1: statistical results of the 21 features identified with postprandial significance (Table A) and with product or age effect (Table B); Figure S1: boxplots of identified serum metabolites with a significant iAUC combined with a product or age effect; Figure S2: relative concentrations of serum metabolites during the 3 week restricted period (V1, V2, V3) and during the 6 h postprandial test after milk and yogurt challenge; Figure S3: mass spectrum of unknown compounds detected in serum samples at levels 3 to 4 in comparison with the mass spectrum of the NIST14 library if possible (PDF)
Table S2. Full data matrix of automatically deconvoluted features of batches 1–3 (XLSX)
Table S3. Full data matrix of the 21 features manually integrated of batches 1–5 (XLSX)

## Abbreviations

DHS-VTT: dynamic headspace vacuum transfer in trap
iAUC: incremental area under the curve
ITEX: in-tube extraction
LOESS: locally estimated scatterplot smoothing signal
GC: gas chromatography
MSD: mass spectrometer detector
MSI: metabolomics standards initiative
OA: older adult
QC: quality control
RI: retention index
RT: retention time
SPE: solid phase extraction
YA: young adult
