# Supplementary material for: Age-Dependent Serum Volatilomics of Milk and Yogurt Intake: A Randomized Crossover Study in Healthy Young and Older Men
Source: J Proteome Res. 2023 Mar 24;22(4):1201–12. doi: 10.1021/acs.jproteome.2c00674 (PMC10088043; doi:10.1021/acs.jproteome.2c00674)
Supplement: Supplementary file 1 — pr2c00674_si_001.pdf [file pr2c00674_si_001.pdf]

Supporting Information

Age-dependent Serum Volatilomics of Milk and Yogurt Intake – A Randomized Crossover Study in Healthy Young and Older Men

Hélène Yi Meng,[1] Jinyoung Kim,[1,2,3] Charlotte Fleuti,[1] Pascal Fuchsmann,[1] Sergio Polakof,[2] Dominique Dardevet,[2] Corinne Marmonier,[3] Kathryn J. Burton-Pimentel,[1] Ulrich Bütikofer,[1] Guy Vergères[1]*

[1]*Agroscope, Schwarzenburgstrasse 161, 3003 Bern, Switzerland*
[2]*Unité de Nutrition Humaine (UNH), INRAE, Université Clermont Auvergne, F-63000 Clermont-Ferrand, France*
[3]*CNIEL, 42 Rue de Châteaudun, F-75009 Paris, France*

*Corresponding author:
Guy Vergères, Agroscope, Schwarzenburgstrasse 161, 3003 Bern, Switzerland, Phone: +41 58 463 81 54, E-mail: guy.vergeres@agroscope.admin.ch

Keywords:
biomarker, volatilomics, VTT extraction, nutrition, plasma serum metabolome, yogurt, milk, age

Table of Contents

Tables:

Page S3: **Table S1.** Statistical results of the 21 features identified with postprandial significance (**Table A**) and with product or age effet (**Table B**).

(Excel File) **Table S2.** Full data matrix of automatically deconvoluted features of batches 1-3.

(Excel File) **Table S3.** Full data matrix after hexanal-d_{12} LOESS normalization of the 21 features manually integrated of batches 1-5.

Figures:

Page S5: **Figure S1.** Boxplots of identified serum metabolites with significant iAUC combined with a product or age effect.

Page S8: **Figure S2.** Relative concentrations of serum metabolites during the 3-week restricted period (V1, V2, V3) and during the 6h postprandial test after milk and yogurt challenge.

Page S19: **Figure S3**. Mass spectrum of unknown compounds detected in serum samples at levels 3 to 4 in comparison with the mass spectrum of the NIST14 library if possible.

Table S1. Statistical results of the 21 features identified with postprandial significance (**Table A**) and with product or age effet (**Table B**). Highlighted in yellow are p-values < 0.05 and in green are FDR < 0.01. Abbreviations: YA, young adults. OA, older adults. M, milk. Y, yogurt. FDR, false discovery rate.

**A**	Restriction phase: V1 vs V4						Postprandial effect											
	ld.f1				wilcoxon		ld.f1								wilcoxon test: iAUC <> 0			
	YA		OA		YA	OA	YA-M		YA-Y		OA-M		OA-Y		Adult Milk	Adult Yogurt	Older Milk	Older Yogurt
	p-value	FDR	p-value	FDR	p-value	p-value	p-value	FDR	p-value	FDR	p-value	FDR	p-value	FDR	p-value	p-value	p-value	p-value
2-methylbutanal	6.16E-02	1.62E-01	5.66E-01	6.61E-01	3.26E-01	6.76E-02	2.68E-04	9.36E-04	9.05E-08	4.75E-07	1.38E-05	4.84E-05	9.08E-07	3.18E-06	8.55E-01	5.42E-01	2.03E-02	2.95E-02
2-methyl-2-butenal	3.33E-01	5.38E-01	3.97E-01	6.61E-01	3.26E-01	1.07E-02	1.85E-04	9.36E-04	1.76E-09	1.23E-08	1.54E-06	6.48E-06	1.33E-11	1.40E-10	7.15E-01	7.35E-01	1.04E-01	1.66E-02
3,5-dimethyloctan-2-one	8.10E-03	4.42E-02	2.12E-03	1.48E-02	5.80E-02	1.22E-04	2.96E-16	6.22E-15	3.87E-16	8.12E-15	4.56E-12	9.58E-11	1.18E-12	2.48E-11	1.22E-04	7.32E-04	2.44E-04	1.22E-04
SM4	8.39E-01	8.41E-01	2.65E-01	6.17E-01	8.08E-01	3.26E-01	5.53E-01	5.53E-01	5.28E-01	5.28E-01	9.07E-02	1.36E-01	1.10E-02	1.45E-02	3.91E-01	1.00E+00	6.76E-02	1.22E-03
3-methyl-2-(5H)-Furanone	5.36E-03	4.42E-02	5.63E-01	6.61E-01	3.58E-01	7.61E-01	1.73E-05	1.21E-04	9.32E-06	2.45E-05	1.01E-03	3.02E-03	1.81E-04	2.71E-04	1.35E-01	1.05E-02	5.02E-01	6.70E-01
2-coumaranone	5.63E-01	7.72E-01	1.79E-02	9.38E-02	6.26E-01	3.05E-03	2.90E-07	3.04E-06	1.28E-09	1.23E-08	1.14E-01	1.59E-01	3.09E-02	3.82E-02	3.66E-04	7.32E-04	4.26E-01	5.42E-01
phenol	2.64E-01	4.62E-01	5.42E-01	6.61E-01	4.63E-01	8.55E-01	3.37E-01	3.73E-01	2.27E-01	2.51E-01	8.55E-10	8.98E-09	2.28E-09	1.60E-08	2.17E-01	3.76E-01	1.22E-04	2.44E-04
p-cresol	8.41E-01	8.41E-01	1.43E-01	4.30E-01	4.26E-01	1.35E-01	1.72E-01	2.25E-01	1.64E-01	2.16E-01	2.42E-07	1.70E-06	2.14E-06	6.42E-06	1.53E-01	4.14E-01	8.54E-04	1.22E-03
acetic acid	8.41E-03	4.42E-02	1.23E-01	4.30E-01	1.53E-01	2.95E-02	4.46E-02	6.24E-02	1.86E-01	2.17E-01	3.11E-01	3.84E-01	5.00E-01	5.00E-01	4.94E-02	8.39E-01	1.94E-01	2.17E-01
2-methylbutanoic acid	1.09E-02	4.58E-02	4.56E-01	6.61E-01	3.26E-01	2.17E-01	1.13E-03	3.38E-03	1.02E-04	1.94E-04	2.09E-03	4.39E-03	3.83E-05	6.71E-05	8.55E-01	3.05E-01	2.45E-02	2.95E-02
3-methylbutanoic acid	3.86E-03	4.42E-02	2.18E-01	5.71E-01	3.91E-01	1.19E-01	2.07E-02	3.35E-02	2.31E-05	4.86E-05	3.54E-02	6.20E-02	3.91E-06	8.52E-06	8.08E-01	9.46E-01	2.95E-02	6.71E-03
cis-2-methyl-2-butenoic acid	4.47E-02	1.34E-01	6.25E-01	6.90E-01	2.17E-01	5.42E-01	2.57E-04	9.36E-04	5.37E-06	1.88E-05	1.72E-03	4.39E-03	4.06E-06	8.52E-06	1.00E+00	1.46E-01	2.95E-02	9.06E-02
3-methyl-2-butenoic acid	3.04E-02	1.06E-01	5.37E-01	6.61E-01	2.96E-01	1.53E-01	1.34E-03	3.52E-03	1.36E-05	3.18E-05	7.60E-02	1.23E-01	2.79E-06	7.33E-06	6.70E-01	8.39E-01	1.94E-01	6.71E-03
trans-2-methyl-2-butenoic acid	8.69E-02	1.95E-01	3.71E-01	6.61E-01	2.41E-01	1.94E-01	2.08E-03	4.84E-03	3.45E-06	1.45E-05	1.29E-02	2.46E-02	2.59E-07	1.09E-06	1.00E+00	1.68E-01	1.19E-01	1.07E-02
octanoic acid	6.24E-01	7.72E-01	1.87E-03	1.48E-02	9.03E-01	2.03E-02	1.35E-02	2.37E-02	7.89E-06	2.37E-05	4.49E-07	2.36E-06	9.67E-09	5.07E-08	2.45E-02	2.44E-01	1.22E-04	6.10E-04
nonanoic acid	6.60E-01	7.72E-01	8.26E-04	1.48E-02	3.91E-01	3.05E-03	3.71E-02	5.57E-02	3.80E-01	3.99E-01	1.36E-01	1.78E-01	1.02E-02	1.43E-02	1.94E-01	2.73E-01	3.58E-01	1.71E-03
decanoic acid	3.94E-01	5.91E-01	5.18E-02	2.18E-01	5.42E-01	4.19E-02	8.36E-03	1.60E-02	1.84E-01	2.17E-01	3.74E-01	4.37E-01	1.11E-04	1.80E-04	4.26E-01	4.97E-01	1.04E-01	8.54E-03
trans-4-octene	6.70E-01	7.72E-01	4.58E-01	6.61E-01	6.70E-01	5.83E-01	2.12E-01	2.61E-01	8.30E-03	1.16E-02	5.32E-01	5.59E-01	1.23E-01	1.36E-01	6.26E-01	1.71E-03	1.00E+00	9.52E-01
trans-2-octene	6.98E-01	7.72E-01	7.39E-01	7.68E-01	5.02E-01	3.91E-01	3.32E-01	3.73E-01	1.95E-03	2.92E-03	6.85E-01	6.85E-01	1.53E-01	1.61E-01	8.55E-01	1.22E-03	9.52E-01	5.83E-01
3-octene	1.49E-01	2.84E-01	7.68E-01	7.68E-01	2.68E-01	2.68E-01	3.98E-01	4.18E-01	6.25E-04	1.01E-03	4.75E-01	5.25E-01	8.83E-02	1.03E-01	8.08E-01	4.88E-04	9.03E-01	5.42E-01
SM21	9.28E-02	1.95E-01	4.66E-01	6.61E-01	4.26E-01	3.58E-01	4.33E-03	9.09E-03	3.12E-04	5.46E-04	1.92E-03	4.39E-03	6.84E-06	1.31E-05	9.52E-01	9.42E-02	7.85E-02	1.66E-02

B	Age or Product effect							
	f1.ld.f1				wilcoxon test			
	Product effect		Age effect		Product effect		Age effect	
	p-value	FDR	p-value	FDR	YA	OA	Milk	Yogurt
2-methylbutanal	8.35E-01	9.08E-01	5.05E-03	8.84E-03	7.87E-01	1.00E+00	5.43E-02	4.27E-02
2-methyl-2-butenal	8.19E-01	9.08E-01	3.30E-02	3.85E-02	9.46E-01	6.70E-01	1.85E-01	4.82E-02
3,5-dimethyloctan-2-one	3.24E-01	8.86E-01	3.85E-01	3.85E-01	5.88E-01	5.02E-01	5.83E-01	1.55E-01
SM4	2.26E-01	8.86E-01	1.30E-03	3.91E-03	7.87E-01	1.35E-01	5.43E-02	1.93E-02
3-methyl-2-(5H)-Furanone	2.04E-01	8.86E-01	1.03E-02	1.44E-02	2.16E-01	9.03E-01	1.05E-01	2.91E-02
2-coumaranone	7.37E-01	9.08E-01	2.06E-03	5.40E-03	6.85E-01	8.55E-01	4.82E-02	4.27E-02
phenol	4.53E-01	8.86E-01	7.53E-06	7.91E-05	1.00E+00	8.55E-01	1.02E-03	3.32E-02
p-cresol	6.78E-01	9.08E-01	7.18E-05	5.02E-04	8.93E-01	4.63E-01	8.23E-04	2.22E-02
acetic acid	4.76E-01	8.86E-01	1.13E-02	1.48E-02	2.44E-01	9.03E-01	9.15E-03	2.39E-01
2-methylbutanoic acid	5.47E-01	8.86E-01	2.87E-03	5.49E-03	3.05E-01	9.03E-01	7.63E-02	1.45E-02
3-methylbutanoic acid	5.48E-01	8.86E-01	7.00E-04	3.16E-03	8.93E-01	7.61E-01	5.43E-02	1.93E-02
cis-2-methyl-2-butenoic acid	3.35E-01	8.86E-01	2.78E-03	5.49E-03	3.05E-01	8.55E-01	6.09E-02	2.91E-02
3-methyl-2-butenoic acid	8.45E-01	9.08E-01	1.93E-02	2.38E-02	4.55E-01	6.26E-01	2.59E-01	3.77E-02
trans-2-methyl-2-butenoic acid	7.49E-01	9.08E-01	2.31E-03	5.40E-03	4.55E-01	6.26E-01	1.69E-01	1.07E-02
octanoic acid	9.16E-01	9.16E-01	2.56E-06	5.38E-05	5.42E-01	8.55E-01	1.07E-02	1.45E-02
nonanoic acid	5.15E-01	8.86E-01	8.51E-04	3.16E-03	6.35E-01	5.42E-01	2.39E-01	9.15E-03
decanoic acid	8.65E-01	9.08E-01	6.32E-03	1.02E-02	6.35E-01	9.03E-01	3.02E-01	2.22E-02
trans-4-octene	1.10E-01	7.72E-01	8.34E-03	1.25E-02	4.79E-02	9.52E-01	5.19E-01	3.95E-03
trans-2-octene	4.87E-02	5.12E-01	5.69E-02	5.98E-02	3.27E-02	9.52E-01	7.93E-01	2.91E-02
3-octene	1.95E-02	4.09E-01	4.24E-02	4.69E-02	2.44E-03	5.02E-01	8.30E-01	1.45E-02
SM21	4.74E-01	8.86E-01	9.02E-04	3.16E-03	3.05E-01	5.83E-01	1.28E-01	2.28E-03

Figure S1. Boxplots of identified serum metabolites with significant iAUC combined with a product or age effect. (A) Boxplot comparison of V1 and V4 in young adults (V1_YA, V4_YA) and older adults (V1_OA, V4_OA). (B) Comparison of postprandial iAUC of young adults after milk or yogurt intake (YA_M and YA_Y) and older adults after milk or yogurt intake (OA_M, OA_Y). The significance for a postprandial effect (iAUC ≠ 0) is shown above each boxplot tests. The significance of the results after Wilcoxon signed rank tests is as follows: *: $p < 0.05$, **: $p < 0.01$, ***: $p < 0.001$, ****: $p < 0.0001$, ns: non-significant (if did not pass ld.f1/f1.ld.f1 tests).

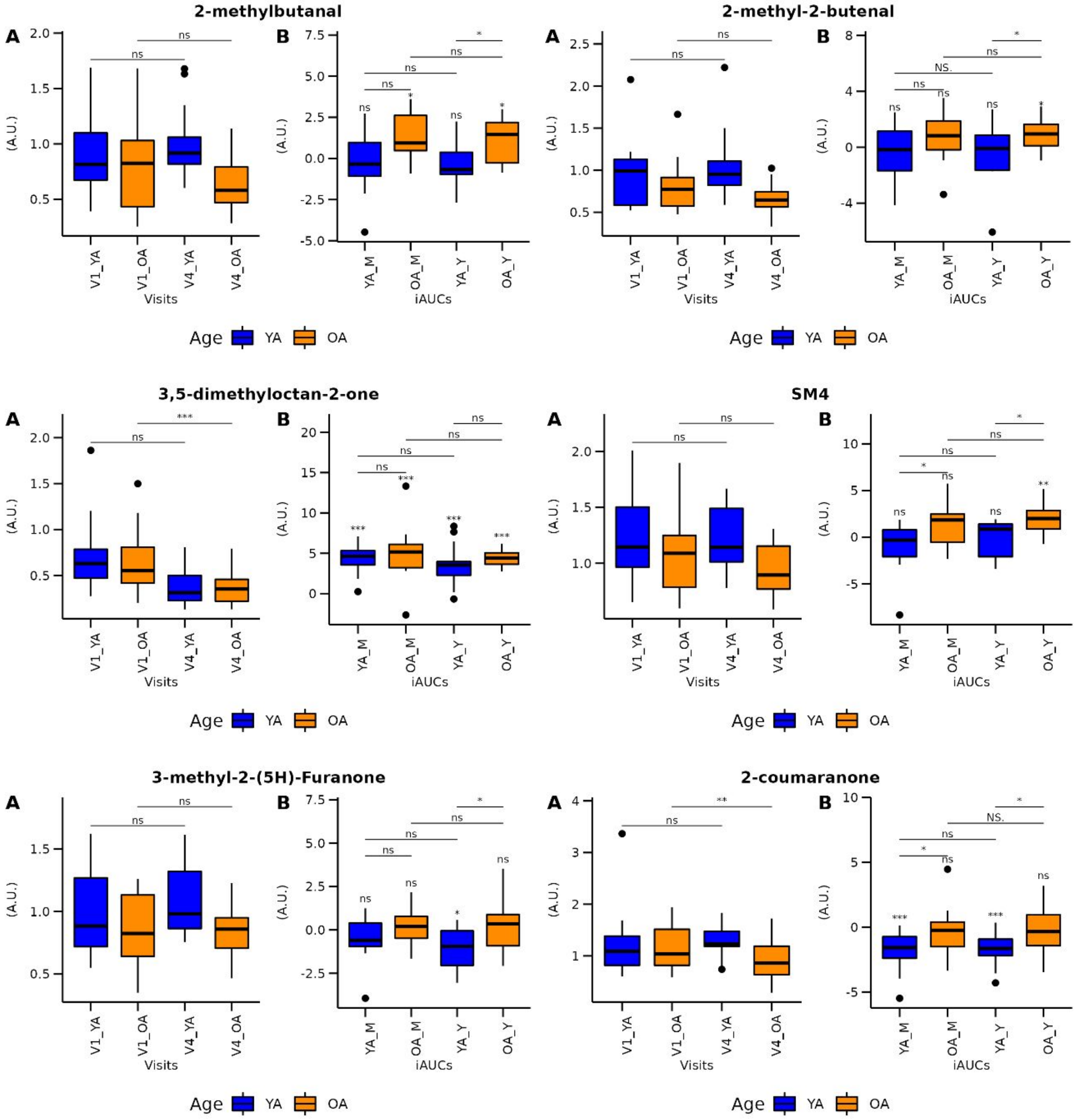

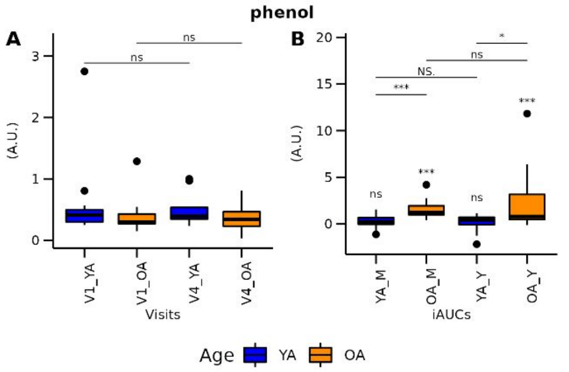
phenol
A
B
(A.U.)
V1_YA
V1_OA
V4_YA
V4_OA
Visits
YA_M
OA_M
YA_Y
OA_Y
iAUCs
Age
YA
OA

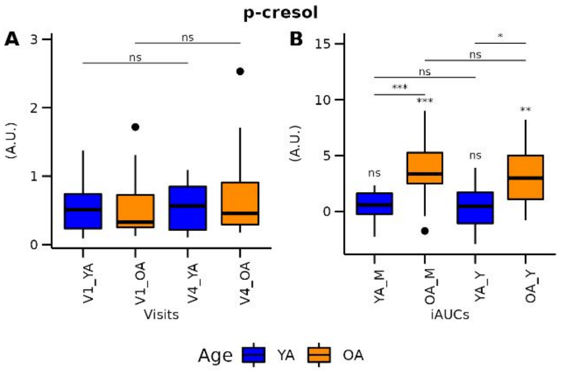
p-cresol
A
B
(A.U.)
V1_YA
V1_OA
V4_YA
V4_OA
Visits
YA_M
OA_M
YA_Y
OA_Y
iAUCs
Age
YA
OA

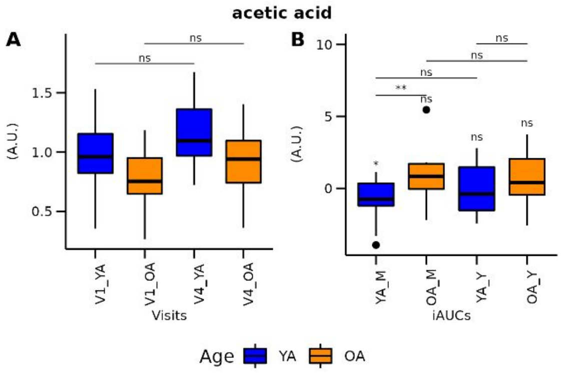
acetic acid
A
B
(A.U.)
V1_YA
V1_OA
V4_YA
V4_OA
Visits
YA_M
OA_M
YA_Y
OA_Y
iAUCs
Age
YA
OA

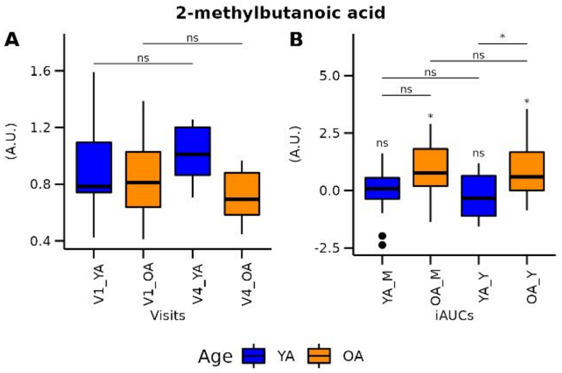
2-methylbutanoic acid
A
B
(A.U.)
V1_YA
V1_OA
V4_YA
V4_OA
Visits
YA_M
OA_M
YA_Y
OA_Y
iAUCs
Age
YA
OA

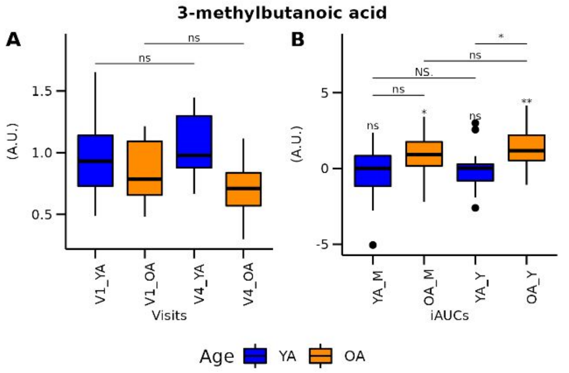
3-methylbutanoic acid
A
B
(A.U.)
V1_YA
V1_OA
V4_YA
V4_OA
Visits
YA_M
OA_M
YA_Y
OA_Y
iAUCs
Age
YA
OA

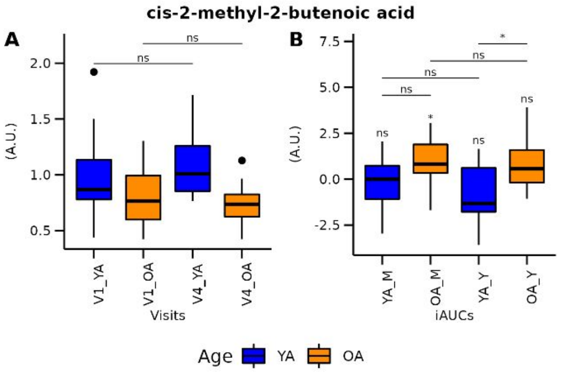
cis-2-methyl-2-butenoic acid
A
B
(A.U.)
V1_YA
V1_OA
V4_YA
V4_OA
Visits
YA_M
OA_M
YA_Y
OA_Y
iAUCs
Age
YA
OA

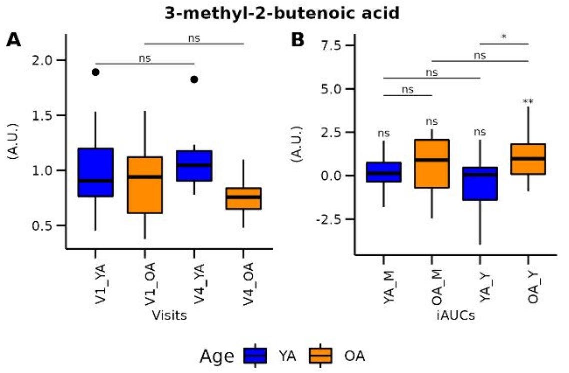
3-methyl-2-butenoic acid
A
B
(A.U.)
V1_YA
V1_OA
V4_YA
V4_OA
Visits
YA_M
OA_M
YA_Y
OA_Y
iAUCs
Age
YA
OA

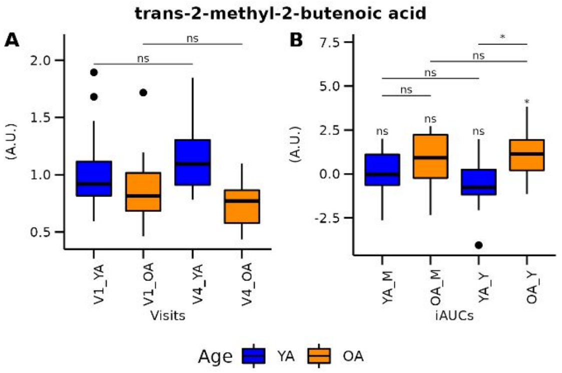
trans-2-methyl-2-butenoic acid
A
B
(A.U.)
V1_YA
V1_OA
V4_YA
V4_OA
Visits
YA_M
OA_M
YA_Y
OA_Y
iAUCs
Age
YA
OA

octanoic acid

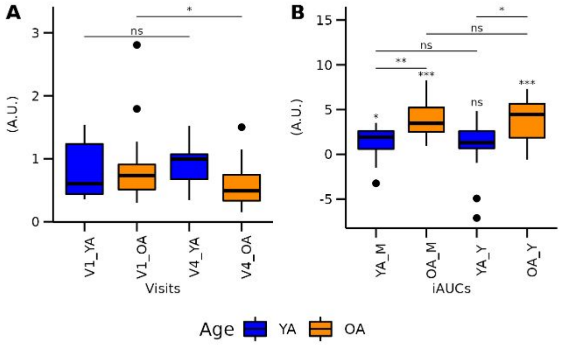

nonanoic acid

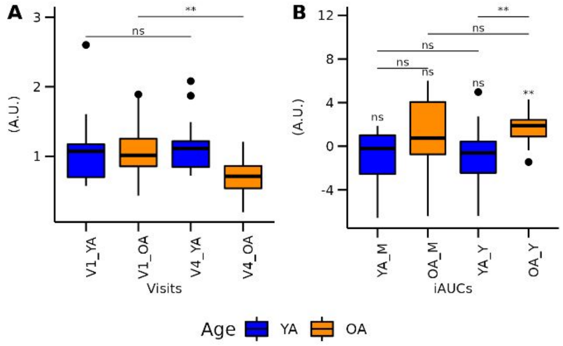

decanoic acid

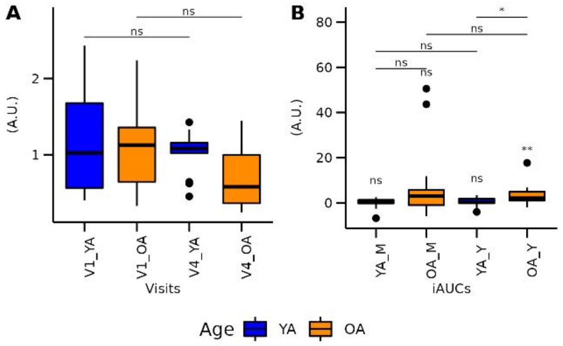

trans-4-octene

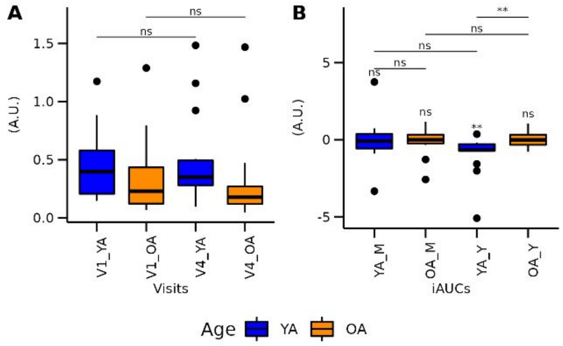

trans-2-octene

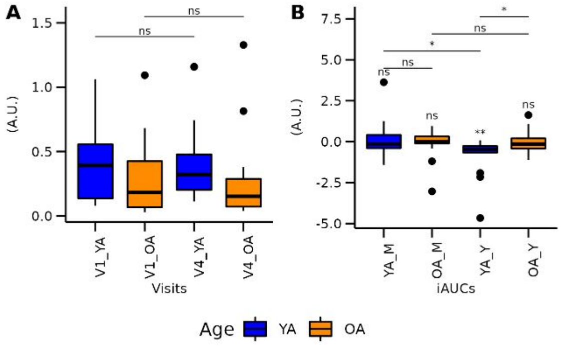

3-octene

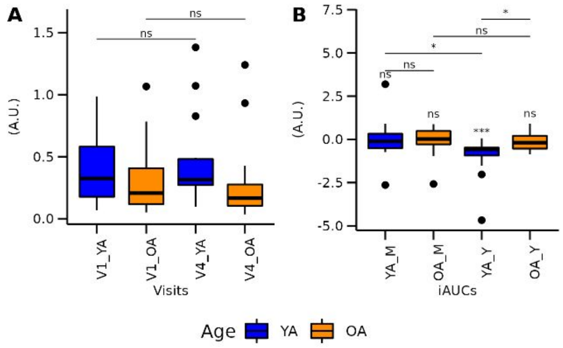

SM21

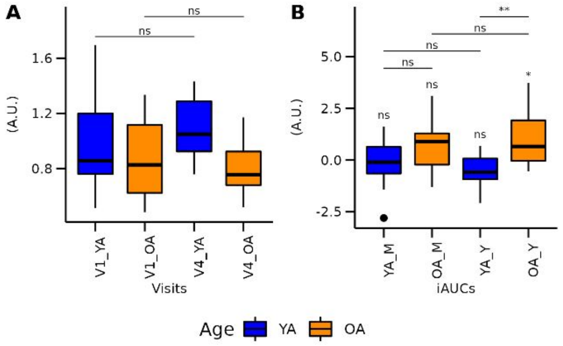

Figure S2. Relative concentrations of serum metabolites during the 3-week restricted period (V1, V2, V3) and during the 6h postprandial test after milk (M0, M15, M30, M60, M90, M120, M180, M240, M300, M360) and yogurt (Y0, Y15, Y30, Y60, Y90, Y120, Y180, Y240, Y300, Y360) challenge. YA: young adults, OA: old adults.

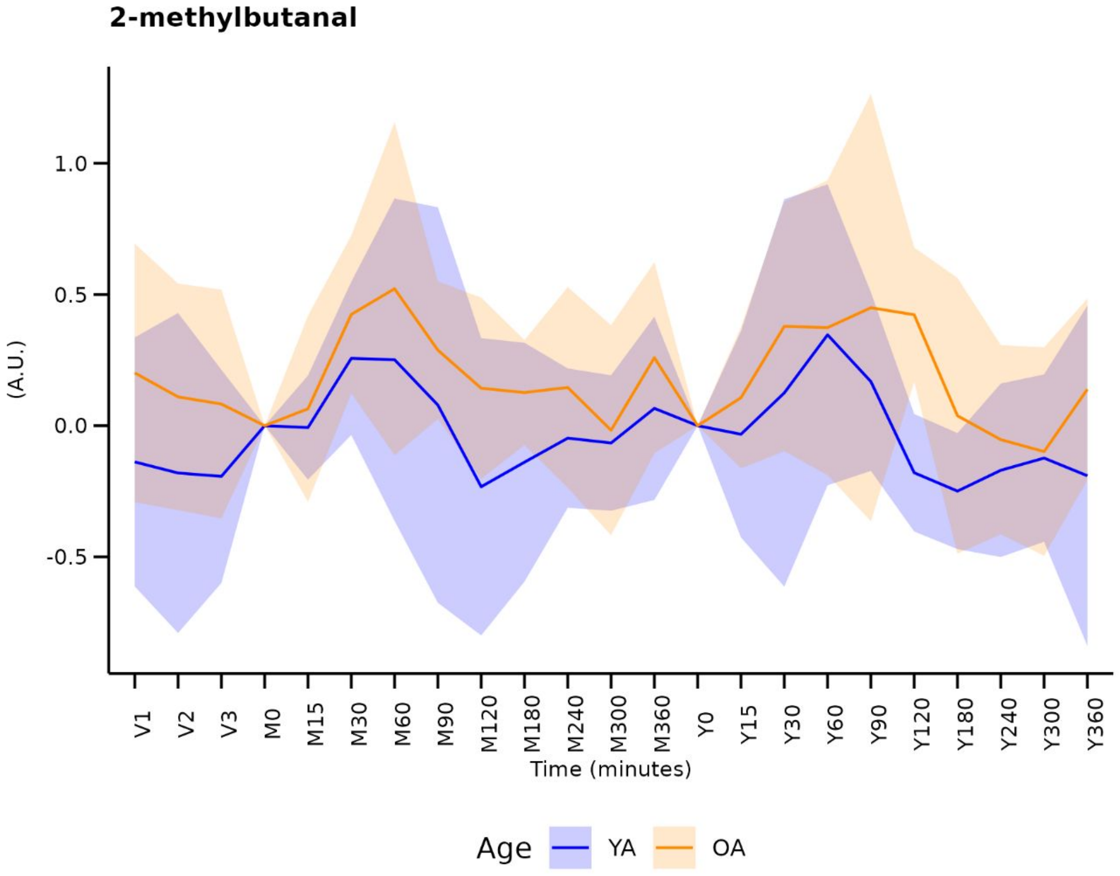

2-methyl-2-butenal

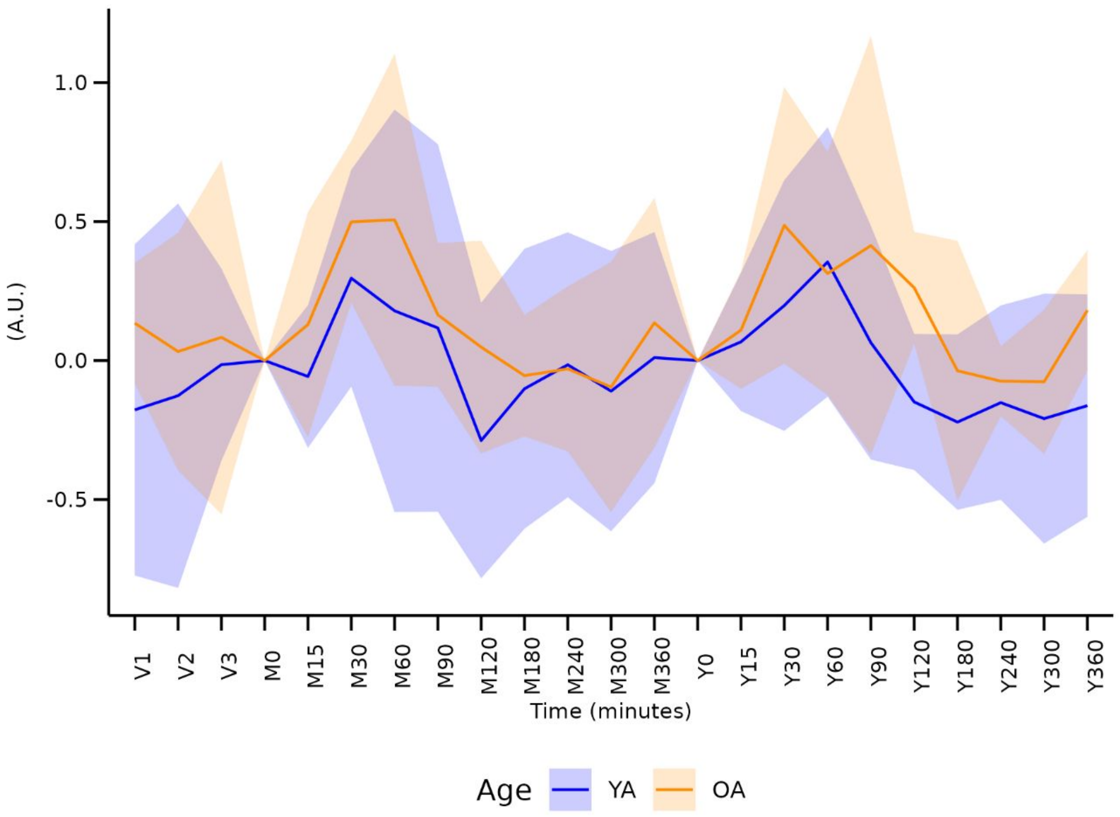

SM4

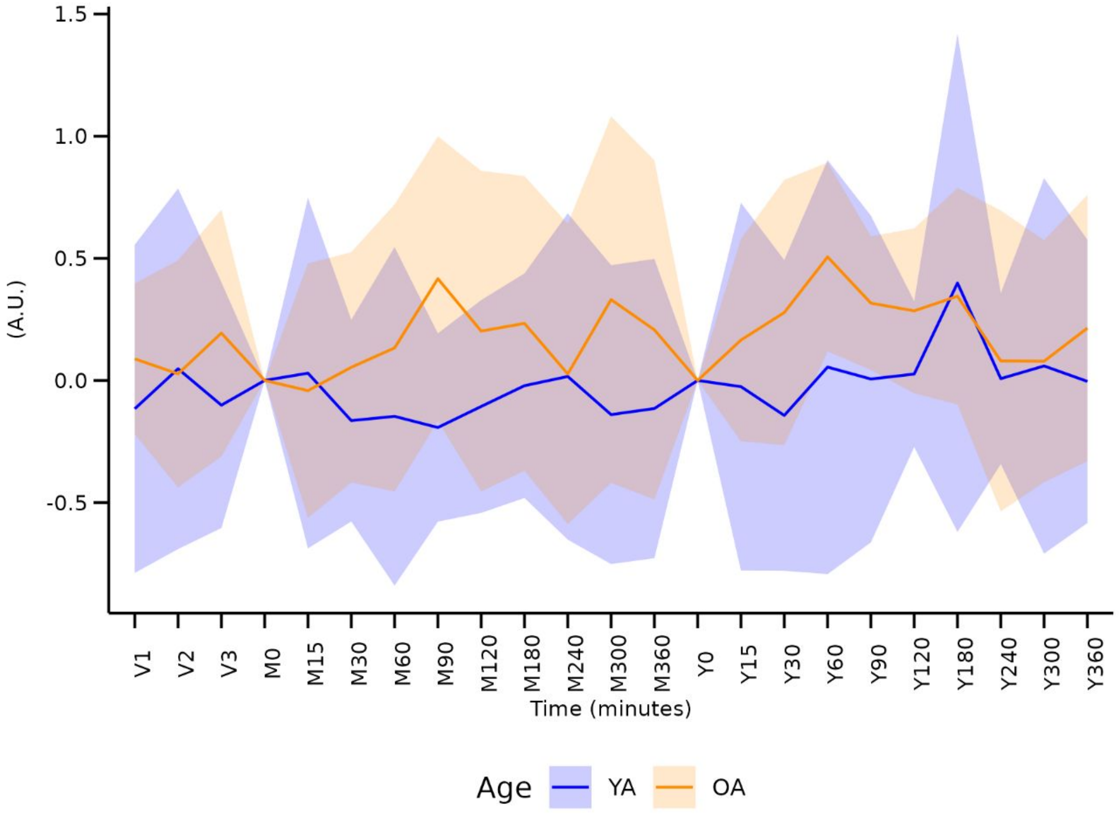

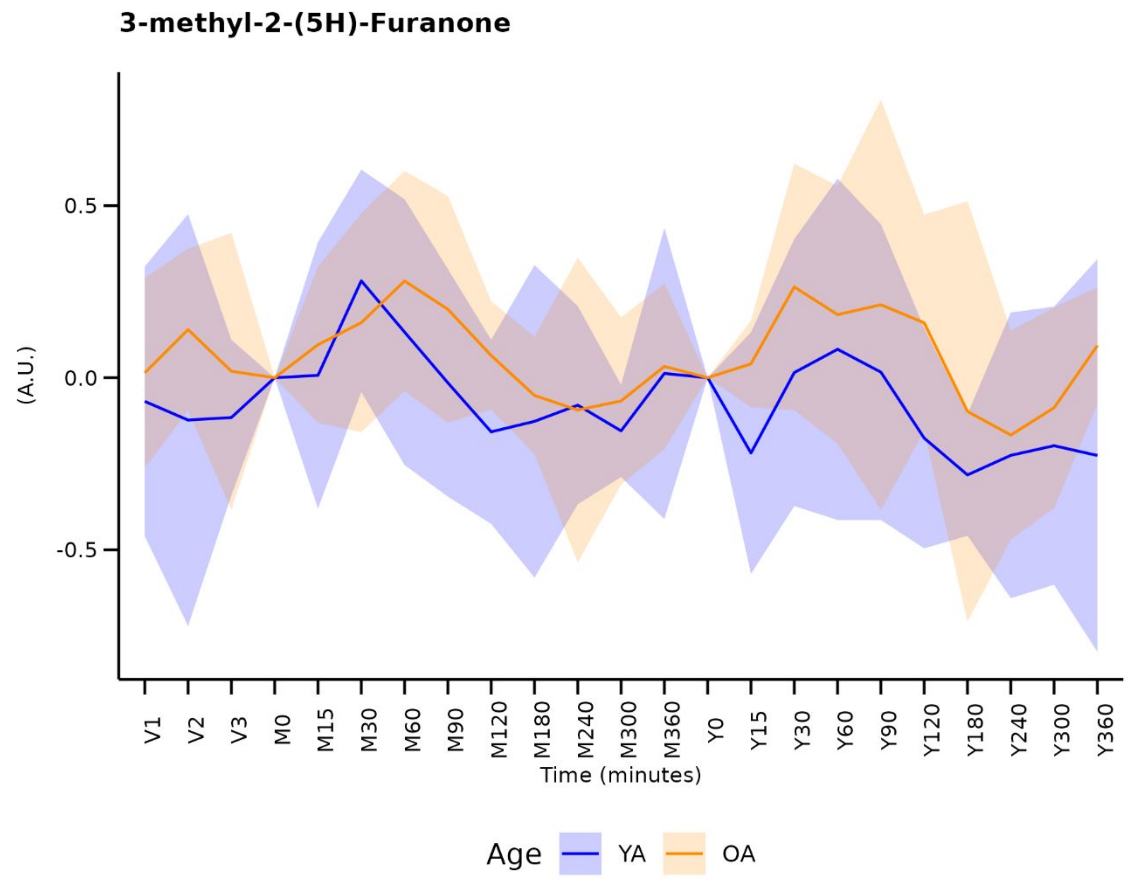

3-methyl-2-(5H)-Furanone
(A.U.)
0.5
0.0
-0.5
V1
V2
V3
M0
M15
M30
M60
M90
M120
M180
M240
M300
M360
Y0
Y15
Y30
Y60
Y90
Y120
Y180
Y240
Y300
Y360
Time (minutes)
Age
YA
OA

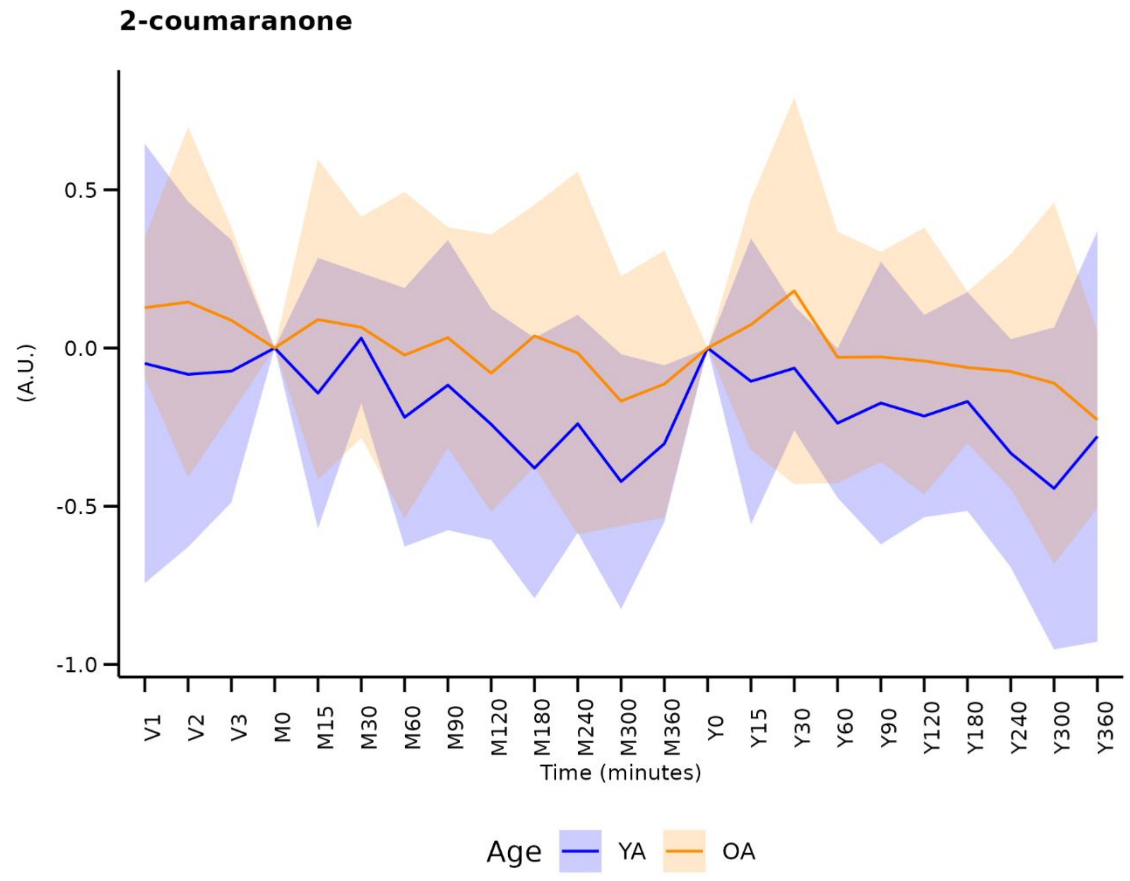

2-coumaranone
(A.U.)
0.5
0.0
-0.5
-1.0
V1
V2
V3
M0
M15
M30
M60
M90
M120
M180
M240
M300
M360
Y0
Y15
Y30
Y60
Y90
Y120
Y180
Y240
Y300
Y360
Time (minutes)
Age
YA
OA

,

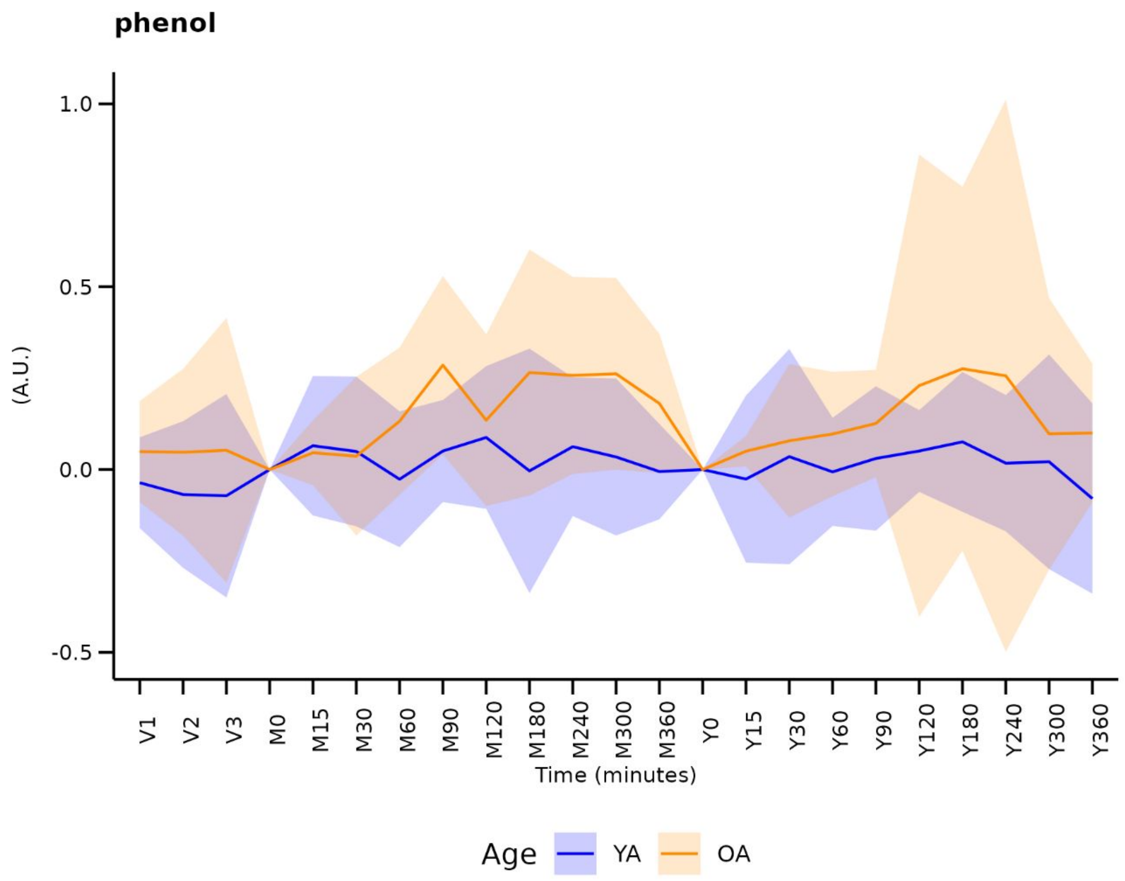
phenol
1.0
0.5
0.0
-0.5
(A.U.)
V1
V2
V3
M0
M15
M30
M60
M90
M120
M180
M240
M300
M360
Y0
Y15
Y30
Y60
Y90
Y120
Y180
Y240
Y300
Y360
Time (minutes)
Age
YA
OA

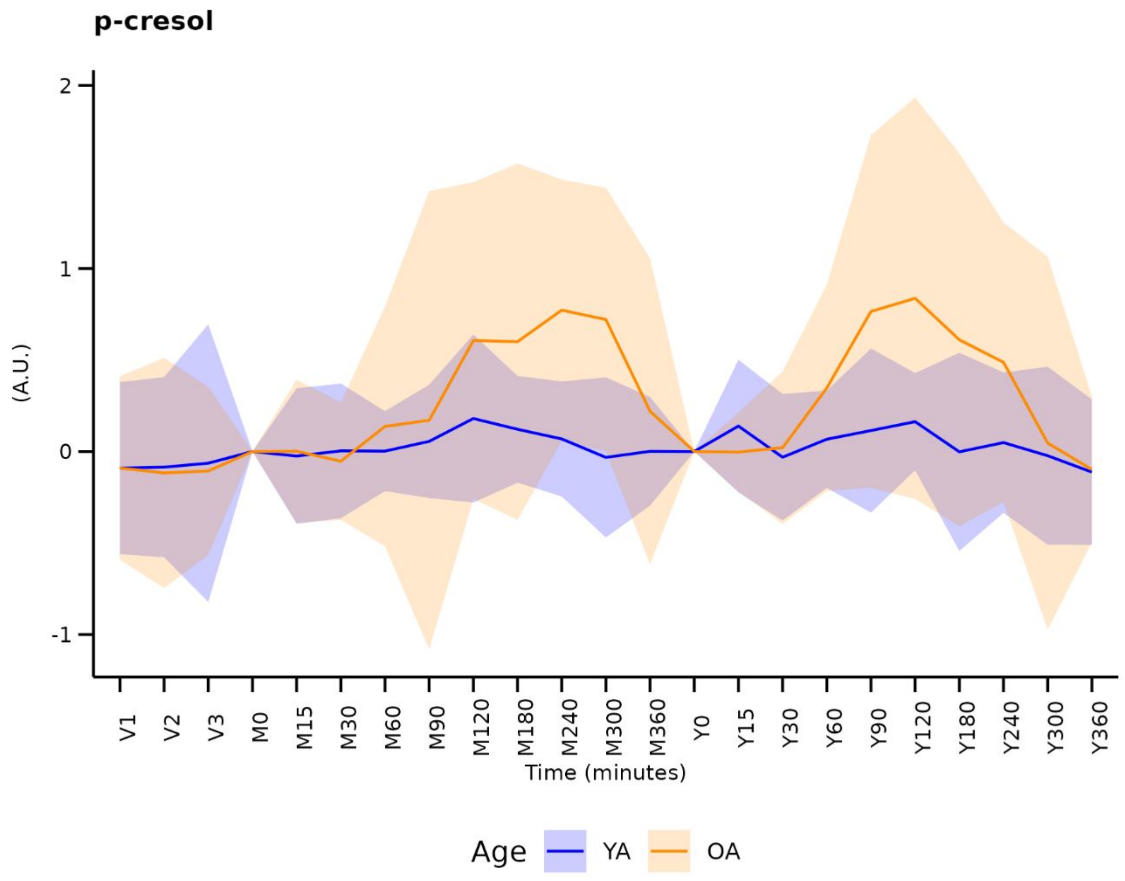
p-cresol
2
1
0
-1
(A.U.)
V1
V2
V3
M0
M15
M30
M60
M90
M120
M180
M240
M300
M360
Y0
Y15
Y30
Y60
Y90
Y120
Y180
Y240
Y300
Y360
Time (minutes)
Age
YA
OA

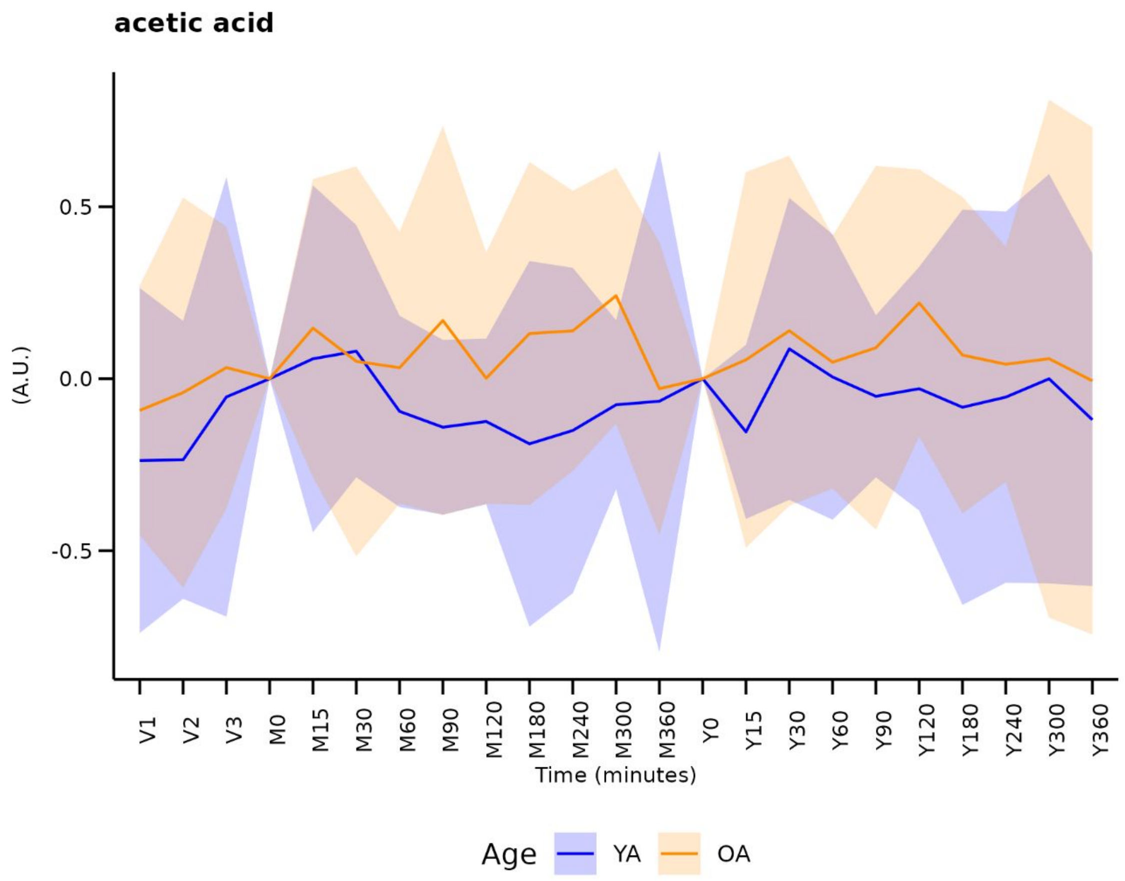
acetic acid
0.5
0.0
-0.5
(A.U.)
V1
V2
V3
M0
M15
M30
M60
M90
M120
M180
M240
M300
M360
Y0
Y15
Y30
Y60
Y90
Y120
Y180
Y240
Y300
Y360
Time (minutes)
Age
YA
OA

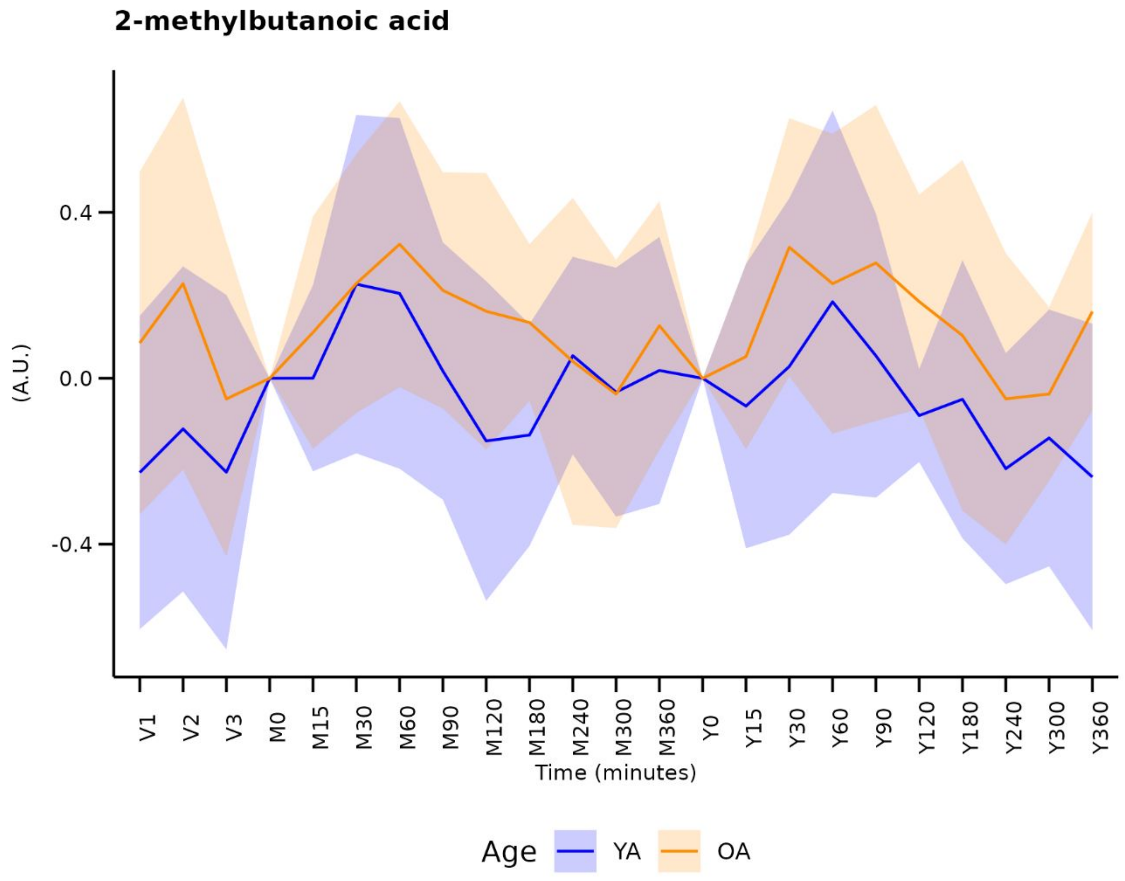
2-methylbutanoic acid
0.4
0.0
-0.4
(A.U.)
V1
V2
V3
M0
M15
M30
M60
M90
M120
M180
M240
M300
M360
Y0
Y15
Y30
Y60
Y90
Y120
Y180
Y240
Y300
Y360
Time (minutes)
Age
YA
OA

3-methylbutanoic acid

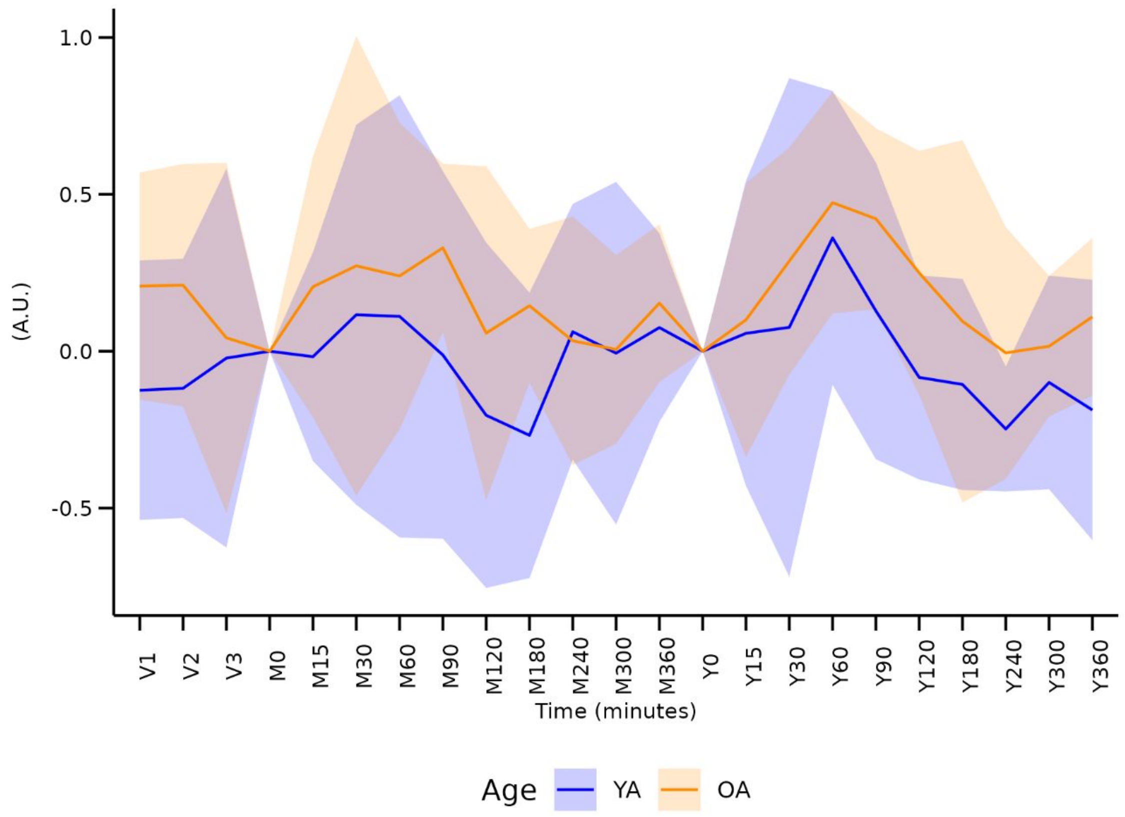

cis-2-methyl-2-butenoic acid

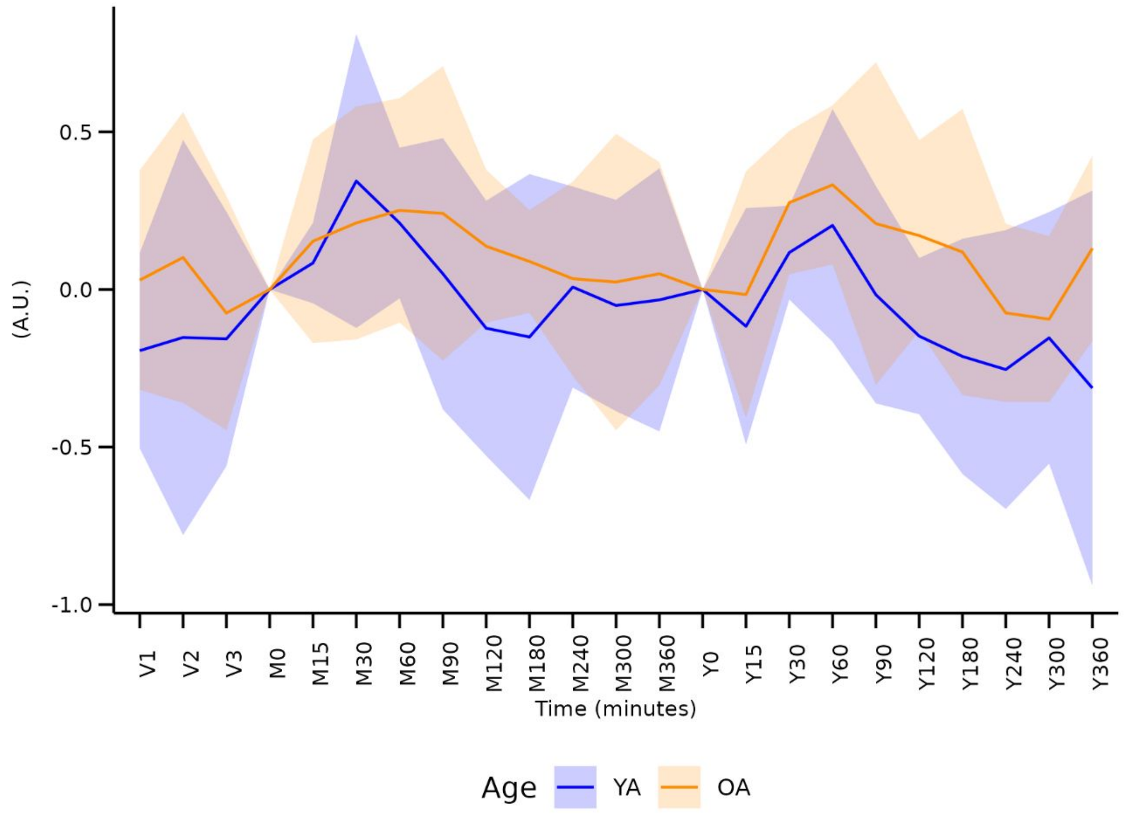

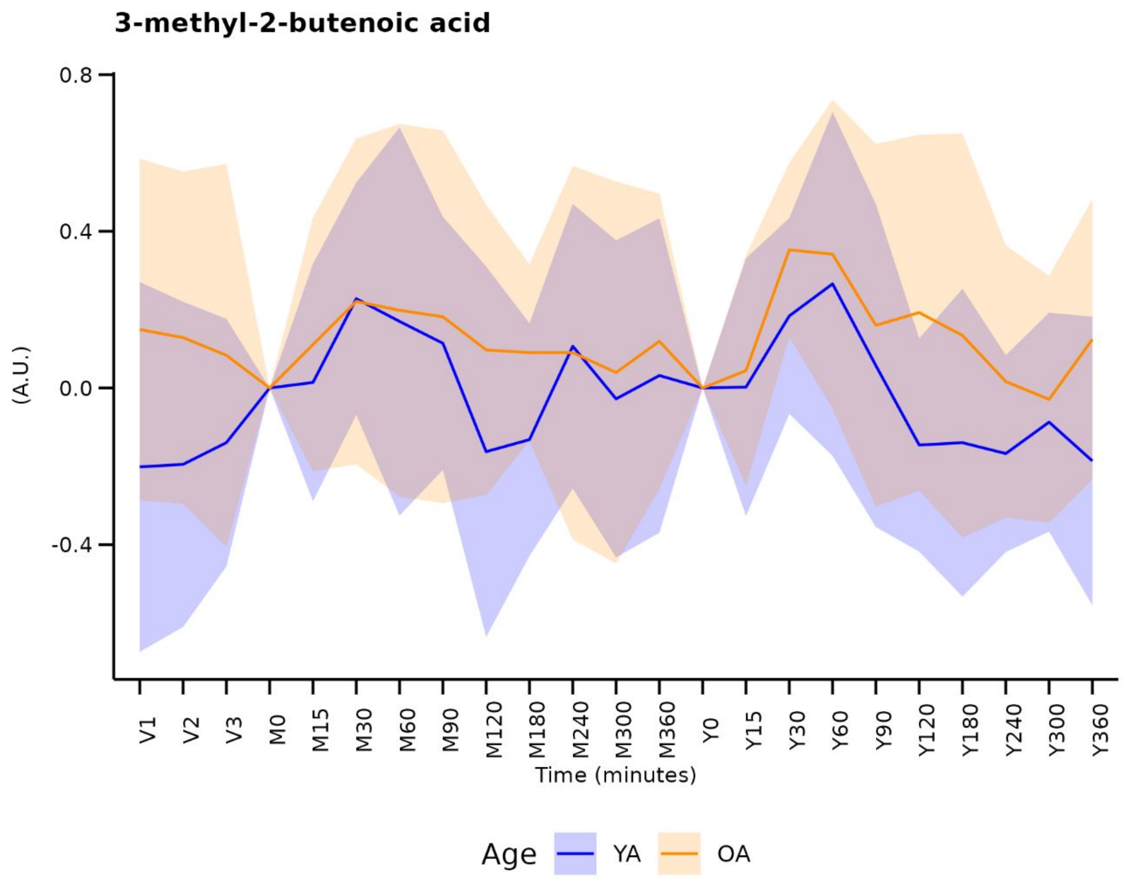
3-methyl-2-butenoic acid
0.8
0.4
0.0
-0.4
(A.U.)
V1
V2
V3
M0
M15
M30
M60
M90
M120
M180
M240
M300
M360
Y0
Y15
Y30
Y60
Y90
Y120
Y180
Y240
Y300
Y360
Time (minutes)
Age
YA
OA

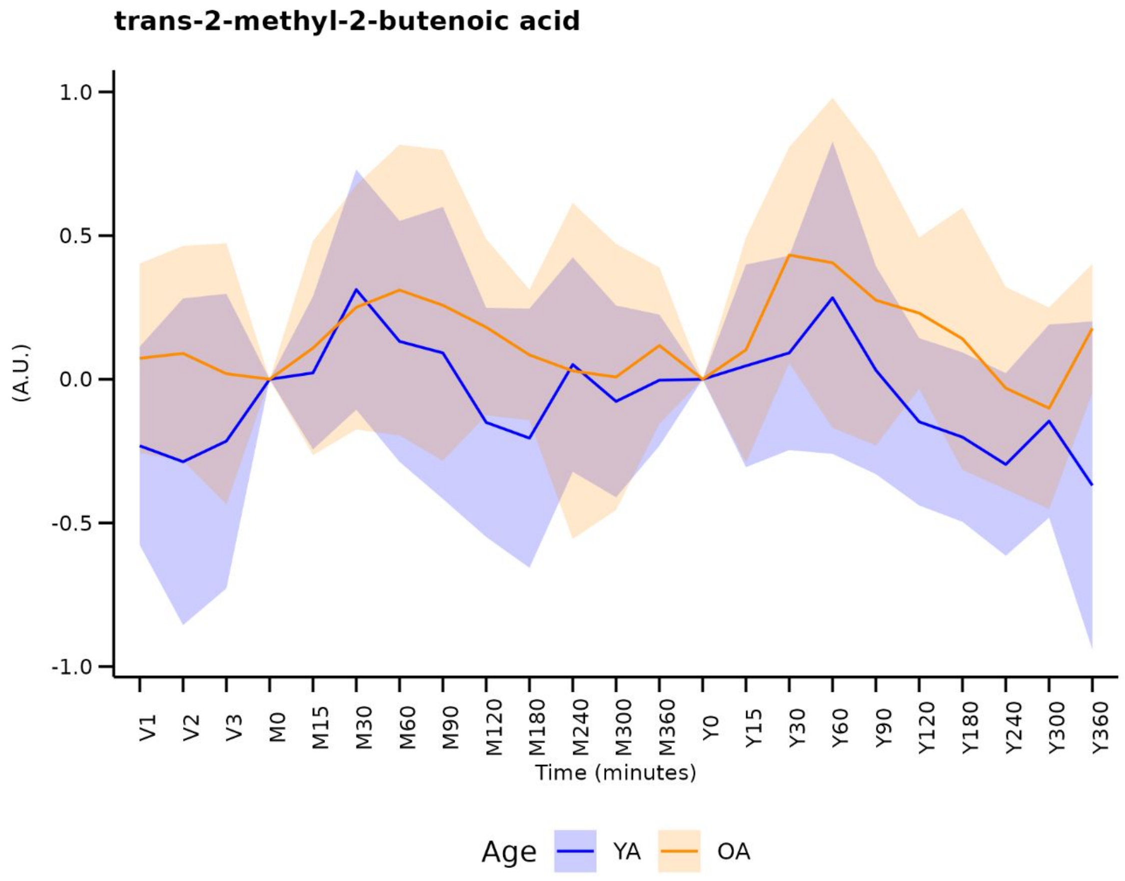
trans-2-methyl-2-butenoic acid
1.0
0.5
0.0
-0.5
-1.0
(A.U.)
V1
V2
V3
M0
M15
M30
M60
M90
M120
M180
M240
M300
M360
Y0
Y15
Y30
Y60
Y90
Y120
Y180
Y240
Y300
Y360
Time (minutes)
Age
YA
OA

octanoic acid

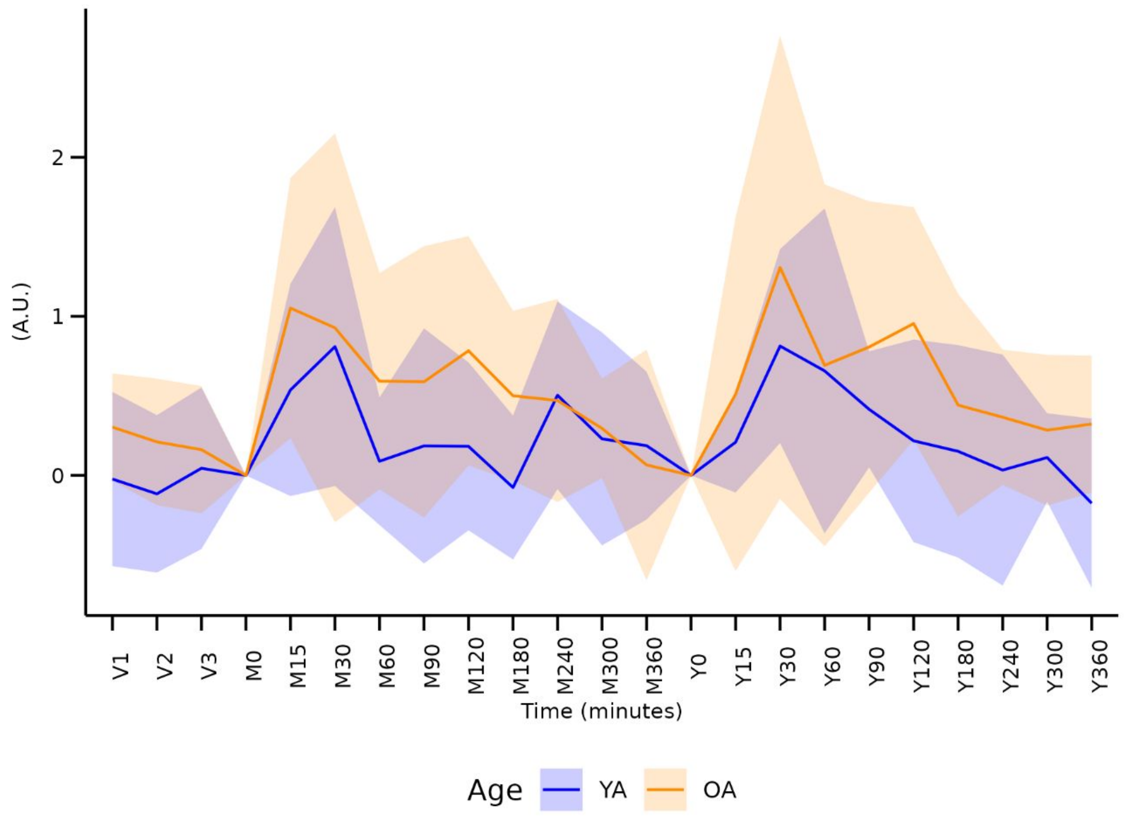

nonanoic acid

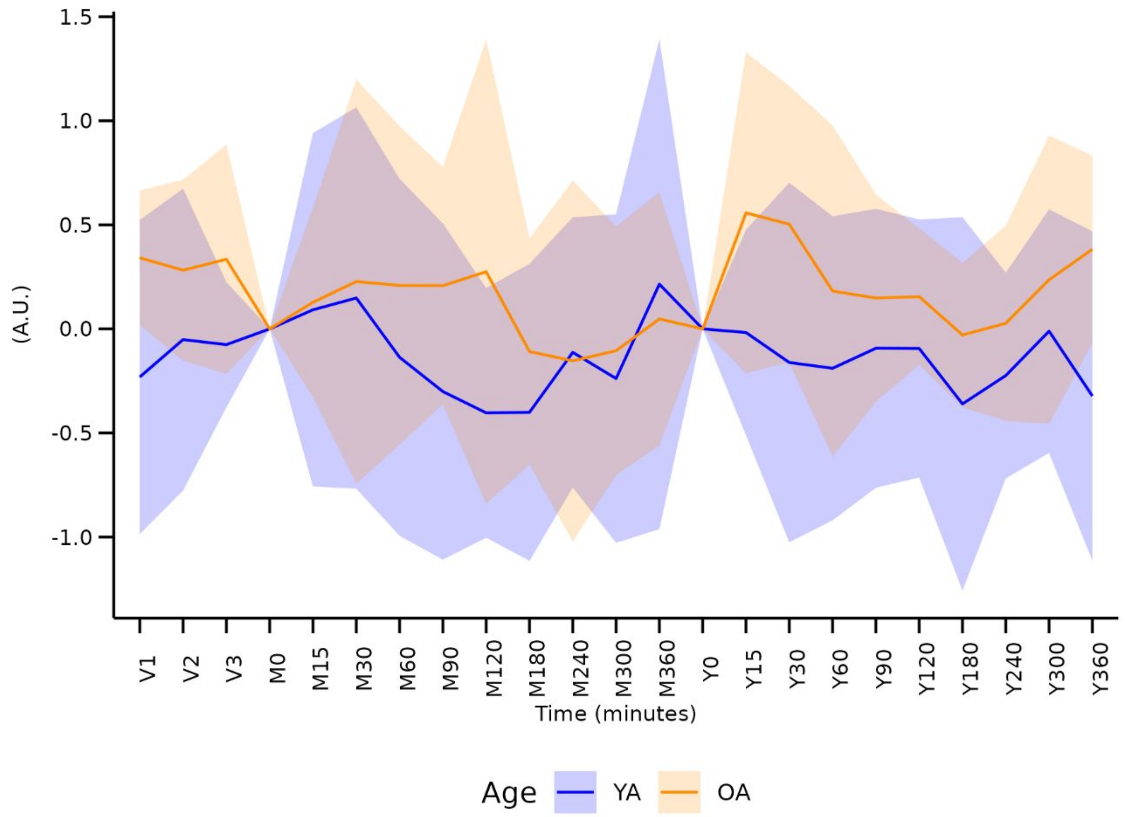

decanoic acid

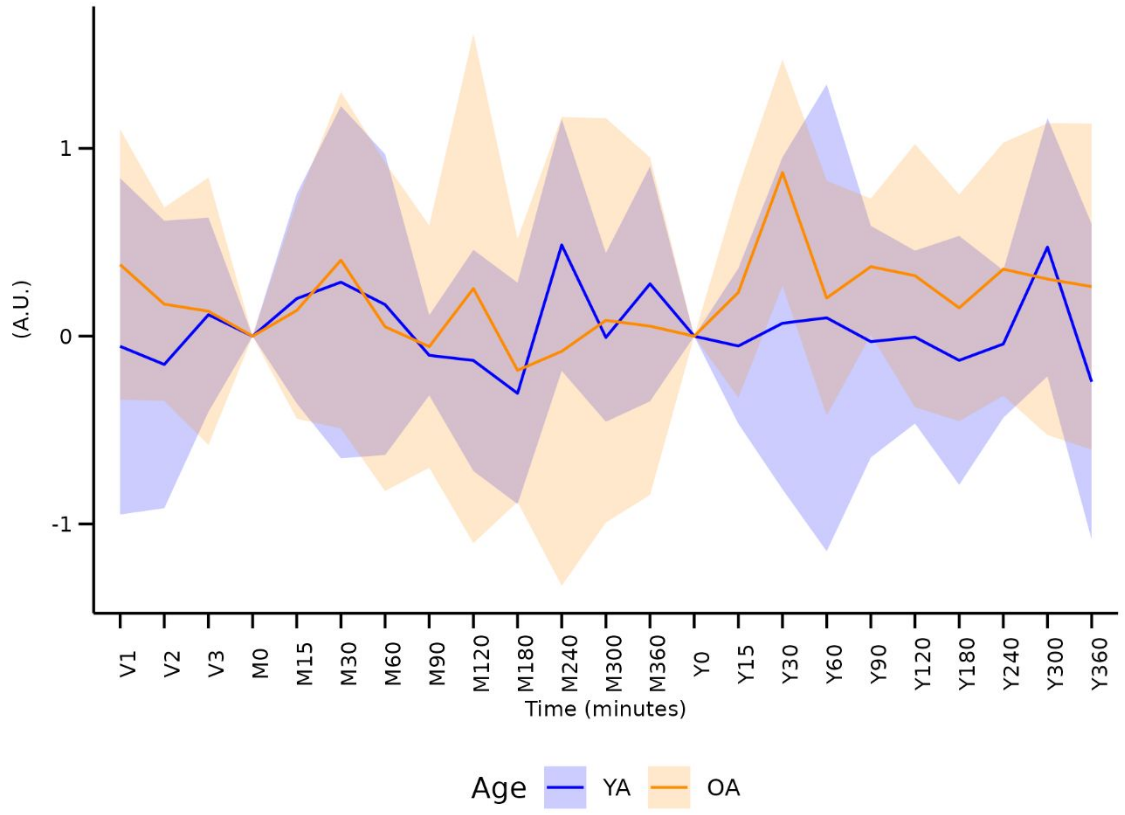

trans-4-octene

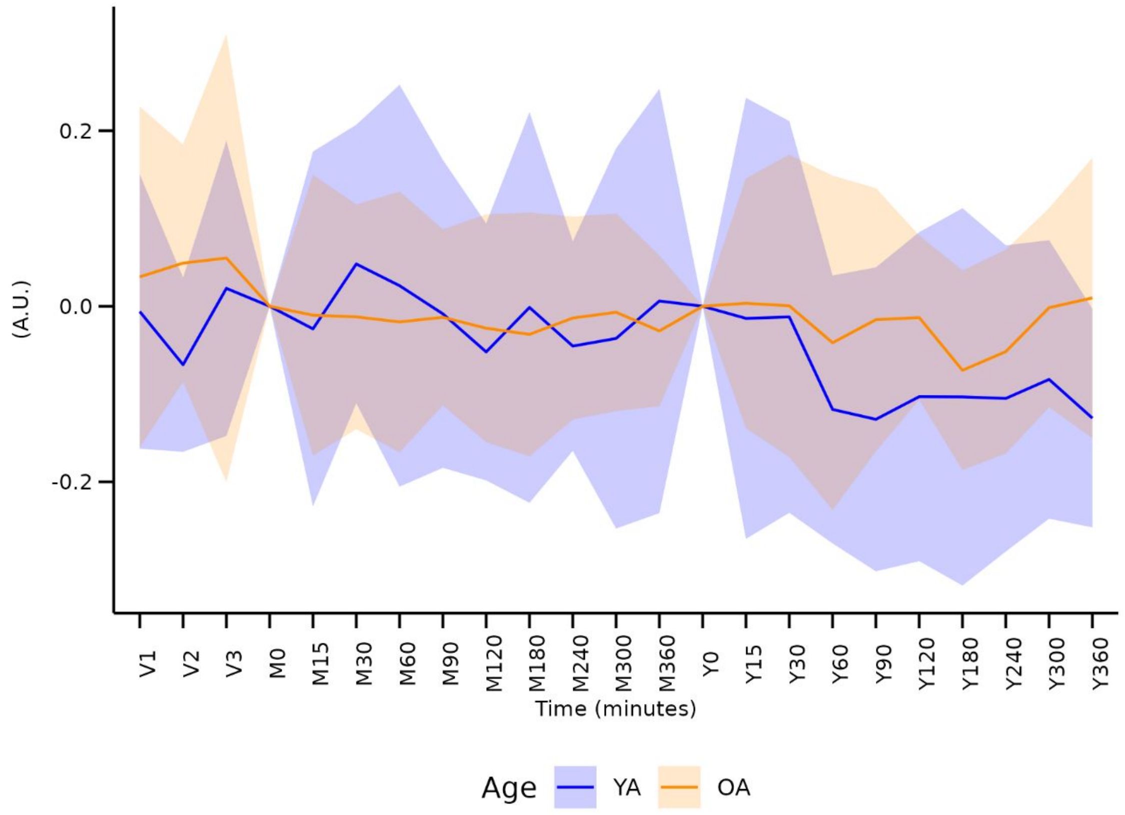

trans-2-octene

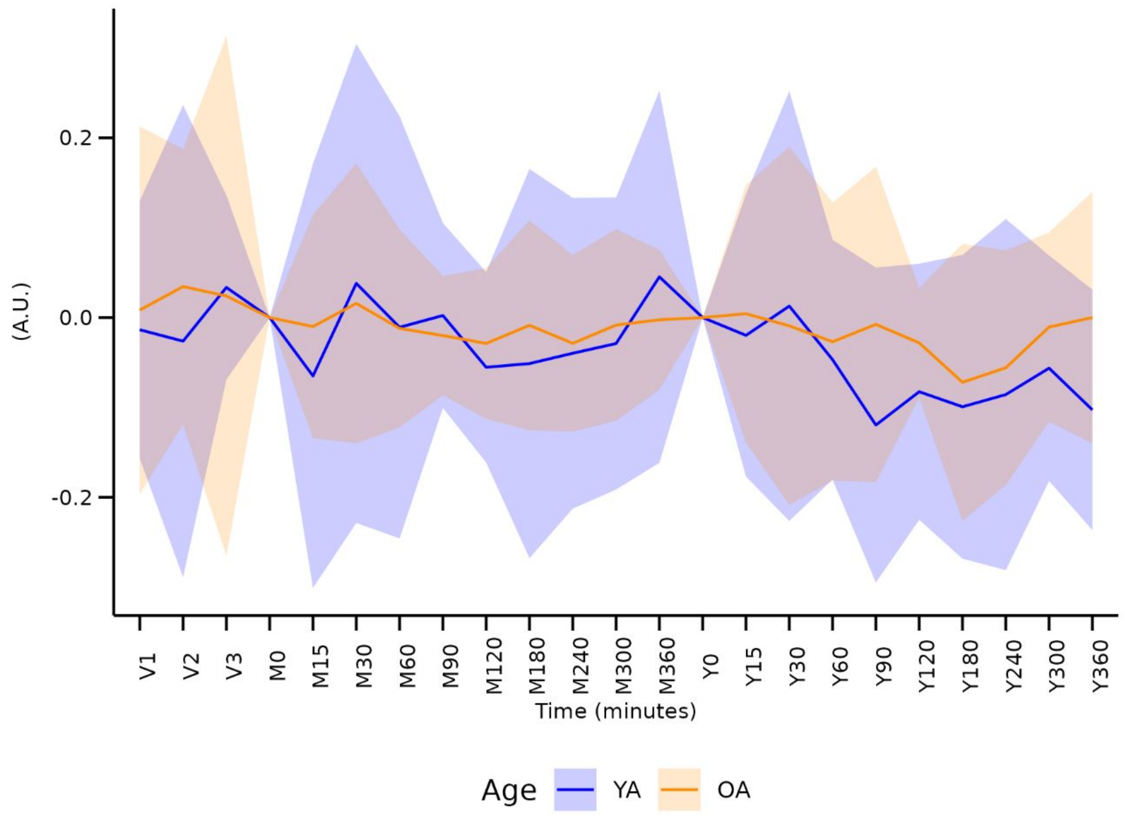

3-octene

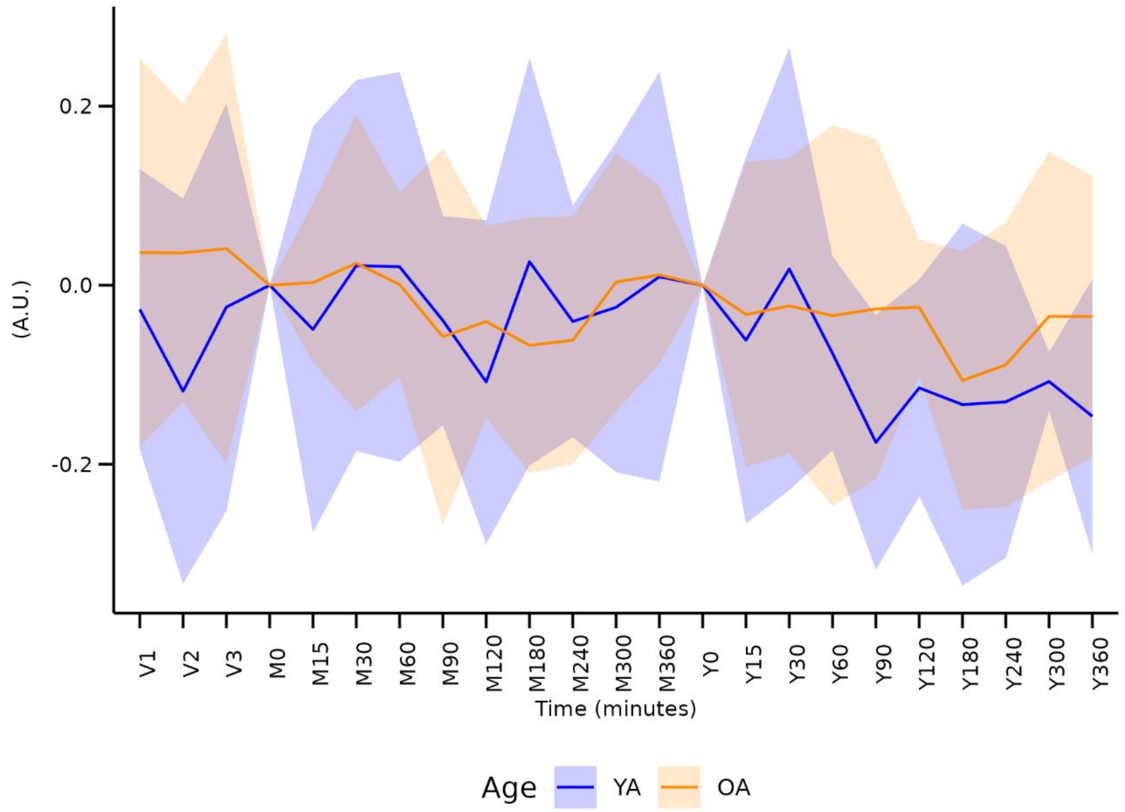

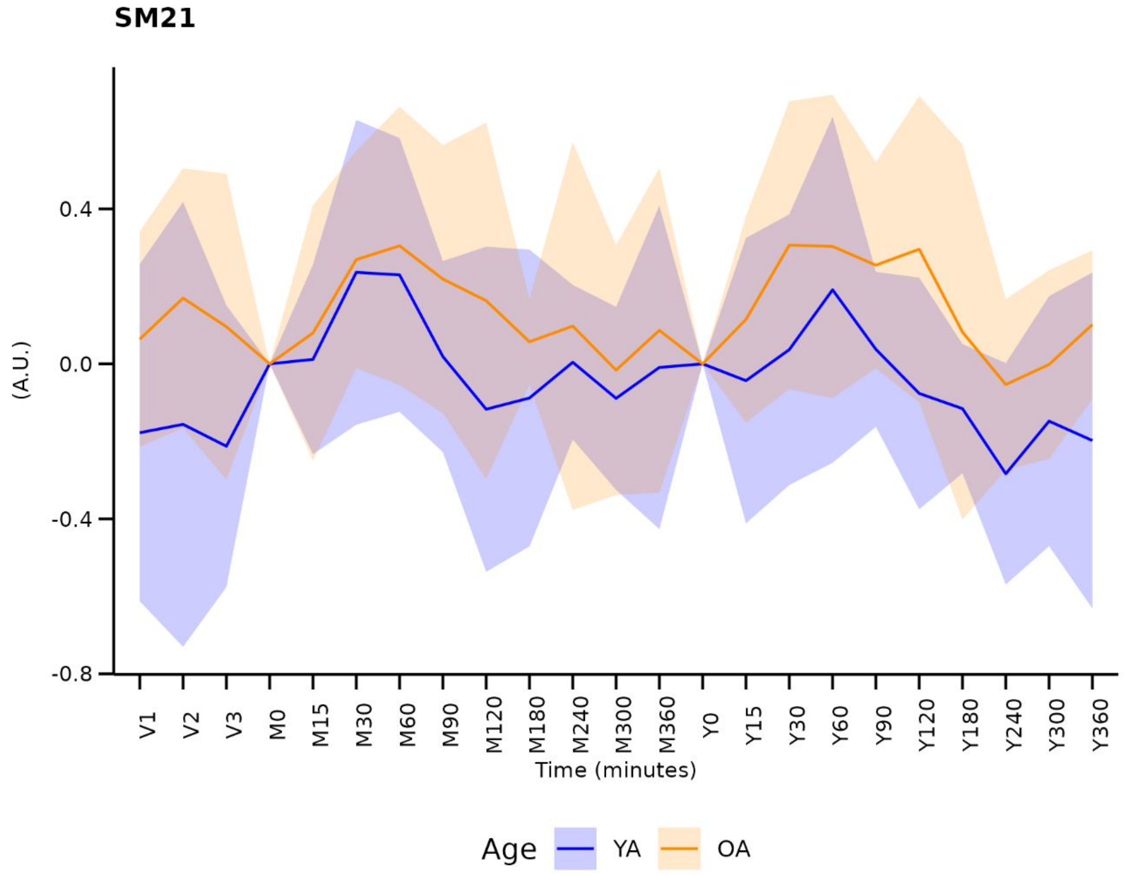
SM21
0.4
0.0
-0.4
-0.8
(A.U.)
V1
V2
V3
M0
M15
M30
M60
M90
M120
M180
M240
M300
M360
Y0
Y15
Y30
Y60
Y90
Y120
Y180
Y240
Y300
Y360
Time (minutes)
Age
YA
OA

Figure S3. Mass spectrum of unknown compounds detected in serum samples at levels 3 to 4 in comparison with the mass spectrum of the NIST14 library if possible.

SM4, RI_{sample} = 1750
Potential NIST identification: 5-ethenyldihydro-5-methyl-2(3H)-Furanone
RI_{lit} = 1683[1], spectral similarity = 82.61%

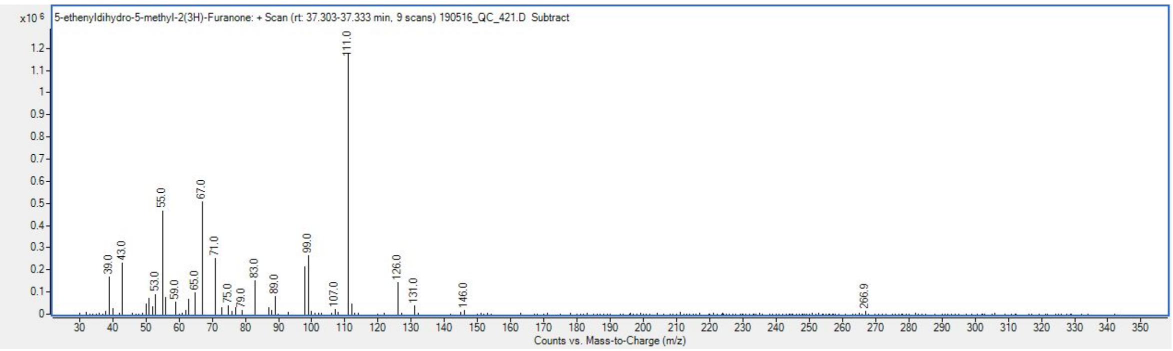

SM21, RI_{sample} = 1695, unknown

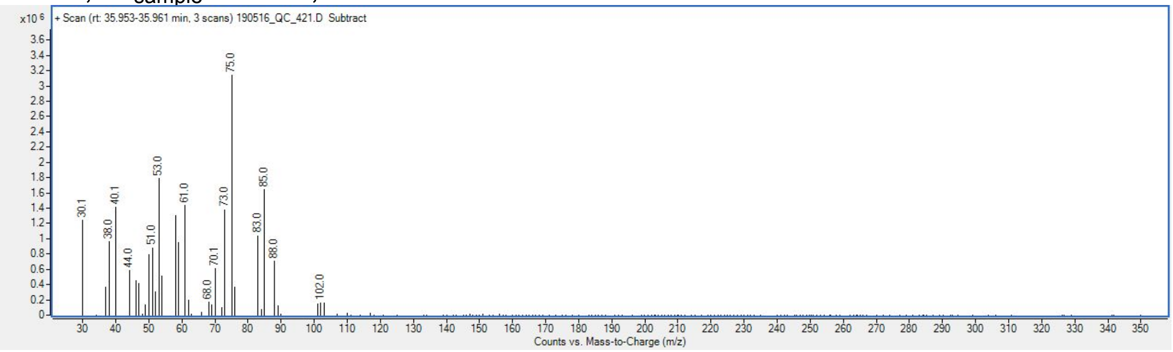

References:

1. Ito, Y.; Sugimoto, A.; Kakuda, T.; Kubota, K., Identification of Potent Odorants in Chinese Jasmine Green Tea Scented with Flowers of Jasminum sambac. *Journal of Agricultural and Food Chemistry* **2002,** 50, (17), 4878-4884.
